# Supplementary figures and images for: Receptor-mediated yolk uptake is required for oskar mRNA localization and cortical anchorage of germ plasm components in the Drosophila oocyte
Source: PLoS Biol. 2021 Apr 23;19(4):e3001183. doi: 10.1371/journal.pbio.3001183 (PMC8064586; doi:10.1371/journal.pbio.3001183)

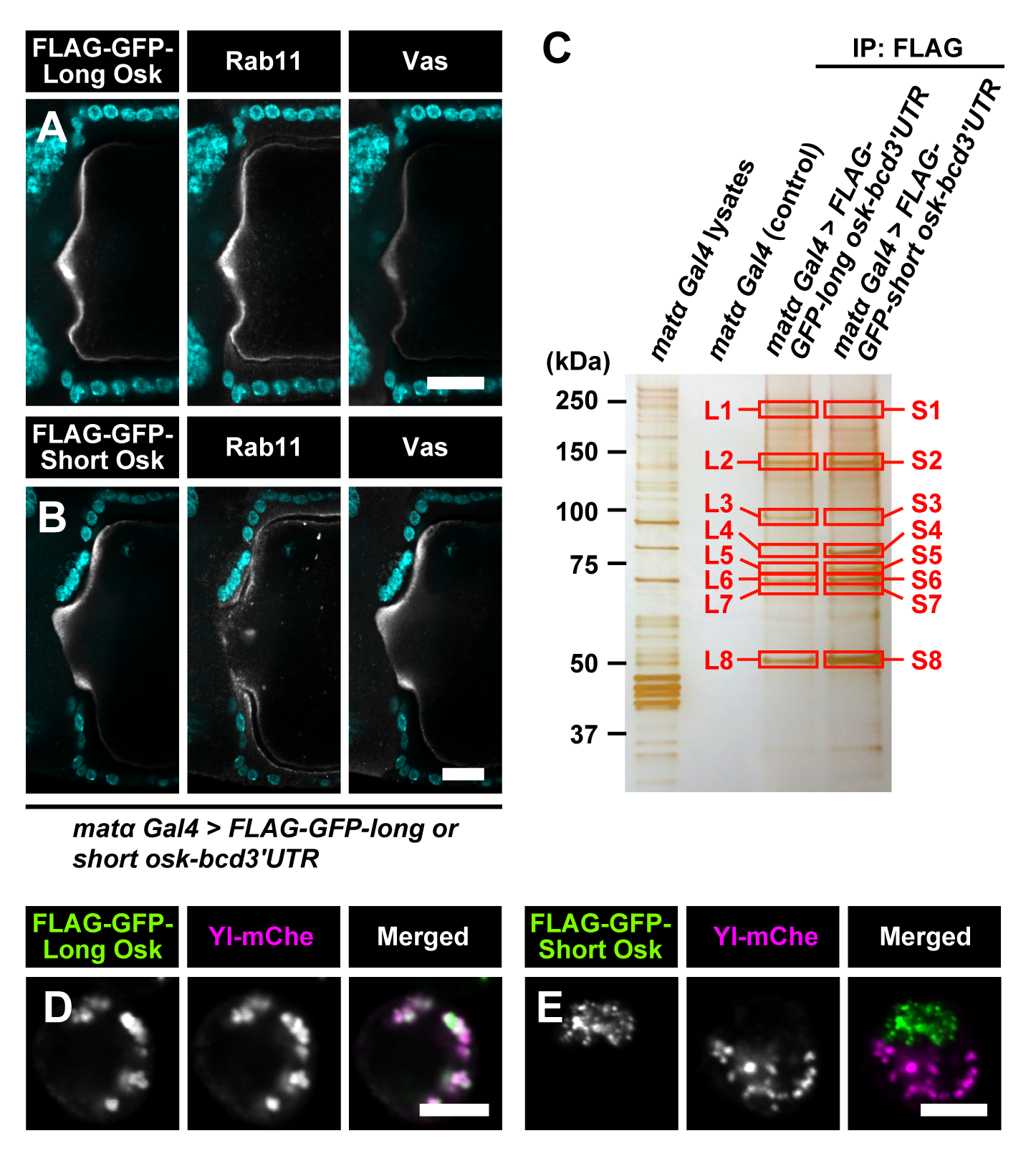

Supplement: S1 Fig — (A, B) Signals for FLAG-GFP-tagged long or short Osk, Rab11, and Vas at the anterior region of the stage 10b oocyte expressing osk-bcd 3′UTR. DNA stained with DPAI is shown in cyan. Endosomal protein Rab11 and germ plasm component Vas were recruited by FLAG-GFP-tagged long and short Osk, respectively, at the anterior region of the oocyte. (C) A silver-stained gel of long or short Osk immunoprecipitates used for the mass spectrometry analysis. Lysates of control oocytes or oocytes expressing 3×FLAG-GFP-long or short Osk were immunoprecipitated using anti-FLAG antibodies. Boxes (labeled L1 to L8 for long Osk IP and S1 to S8 for short Osk IP) represent protein bands that were excised for the mass spectrometry analysis. (D, E) Localization of 3×FLAG-GFP-long or short Osk and Yl-mChe in S2 cells. S2 cells were cotransfected with plasmids that express 3×FLAG-GFP-long or short Osk and Yl-mChe. An uncropped gel image for panel C can be found in S1 Raw Images. Scale bars: 20 μm (A, B) or 5 μm (D, E). GFP, green fluorescent protein; IP, immunoprecipitation; mChe, mCherry; Osk, Oskar; Vas, Vasa; Yl, Yolkless. (TIF) [file pbio.3001183.s001.tif]

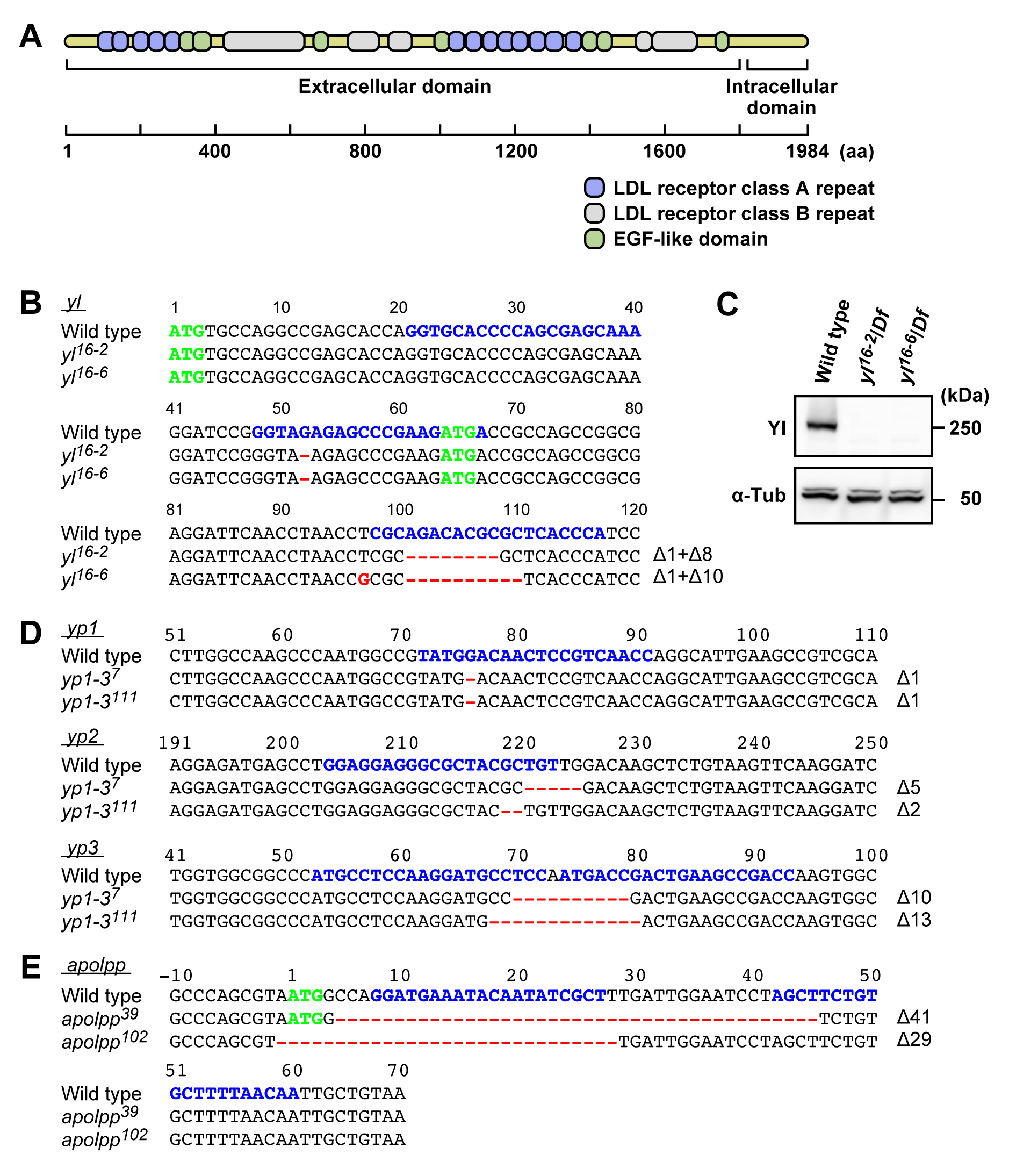

Supplement: S2 Fig — (A) A schematic drawing of domain organization of Yl protein. Blue, gray, and green boxes indicate LDL receptor class A repeat, LDL receptor class B repeat, and EGF-like domain, respectively. (B) DNA sequences of yl gene of wild type and 2 yl alleles (yl16-2 and yl16-6). Target sites for sgRNA are shown in blue. The deletion and point mutation are shown in red. An annotated ATG, which does not match to canonical translation initiation consensus, and a downstream in-frame second ATG are shown in green. Numbering shown above DNA sequences is started with number 1 at the A of the initiation ATG codon. (C) Immunoblots for Yl of ovarian lysates from wild-type, hemizygous yl16-2/Df(1)KA9, and yl16-6/Df(1)KA9 females. The protein band of Yl at about 250 kDa was undetectable in yl-deficient mutants. α-Tub was used as a loading control. (D, E) DNA sequences of yp1, yp2, yp3 (D), and apolpp (E) genes around the mutation sites. Target sites for sgRNA are shown in blue. Deleted bases are shown as red dashes. The ATG corresponding to translational initiation site is shown in green. Numbering shown above DNA sequences is started with number 1 at the A of the initiation ATG codon. Uncropped blot images for panel B can be found in S1 Raw Images. α-Tub, α-Tubulin; EGF, epidermal growth factor; LDL, low-density lipoprotein; sgRNA, single guide RNA; Yl, Yolkless. (TIF) [file pbio.3001183.s002.tif]

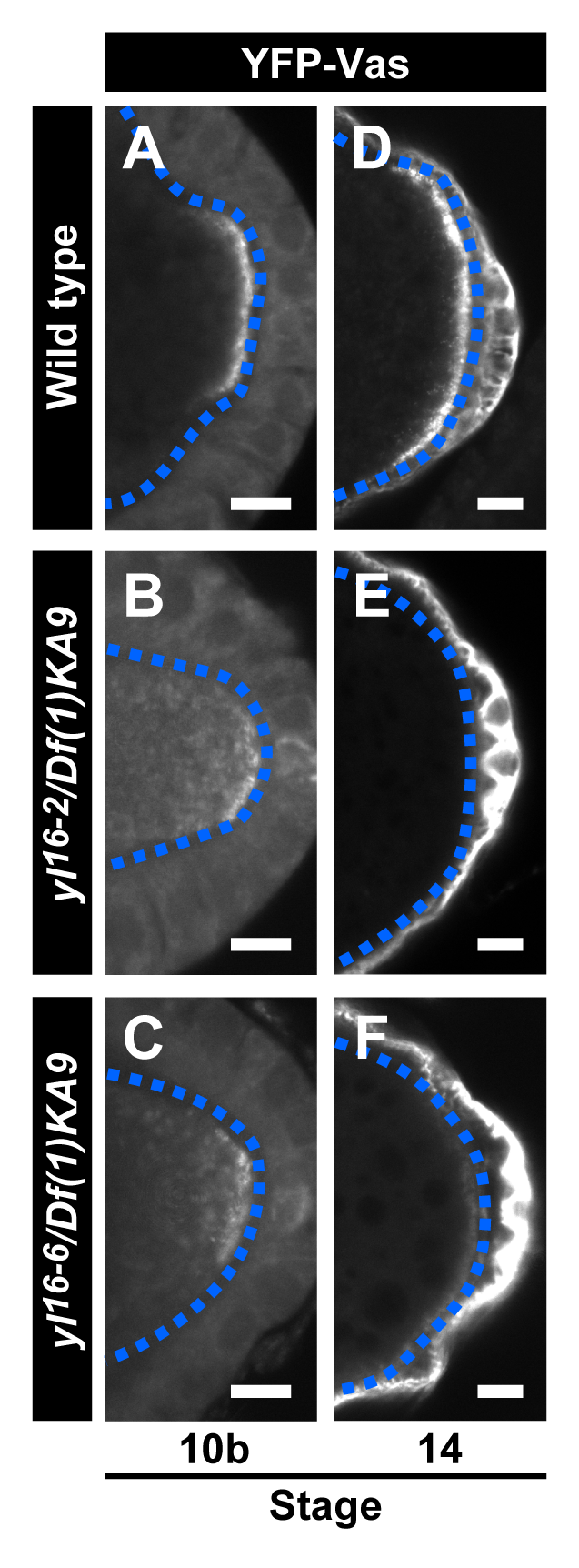

Supplement: S3 Fig — Localization of YFP-Vas at the posterior region of oocytes in wild type (A, D), yl16-2/Df(1)KA9 (B, E), and yl16-6/Df(1)KA9 (C, F) at stages 10b and 14. Oocytes are outlined by blue dashed lines. Scale bars: 10 μm. Vas, Vasa; YFP, yellow fluorescent protein. (TIF) [file pbio.3001183.s003.tif]

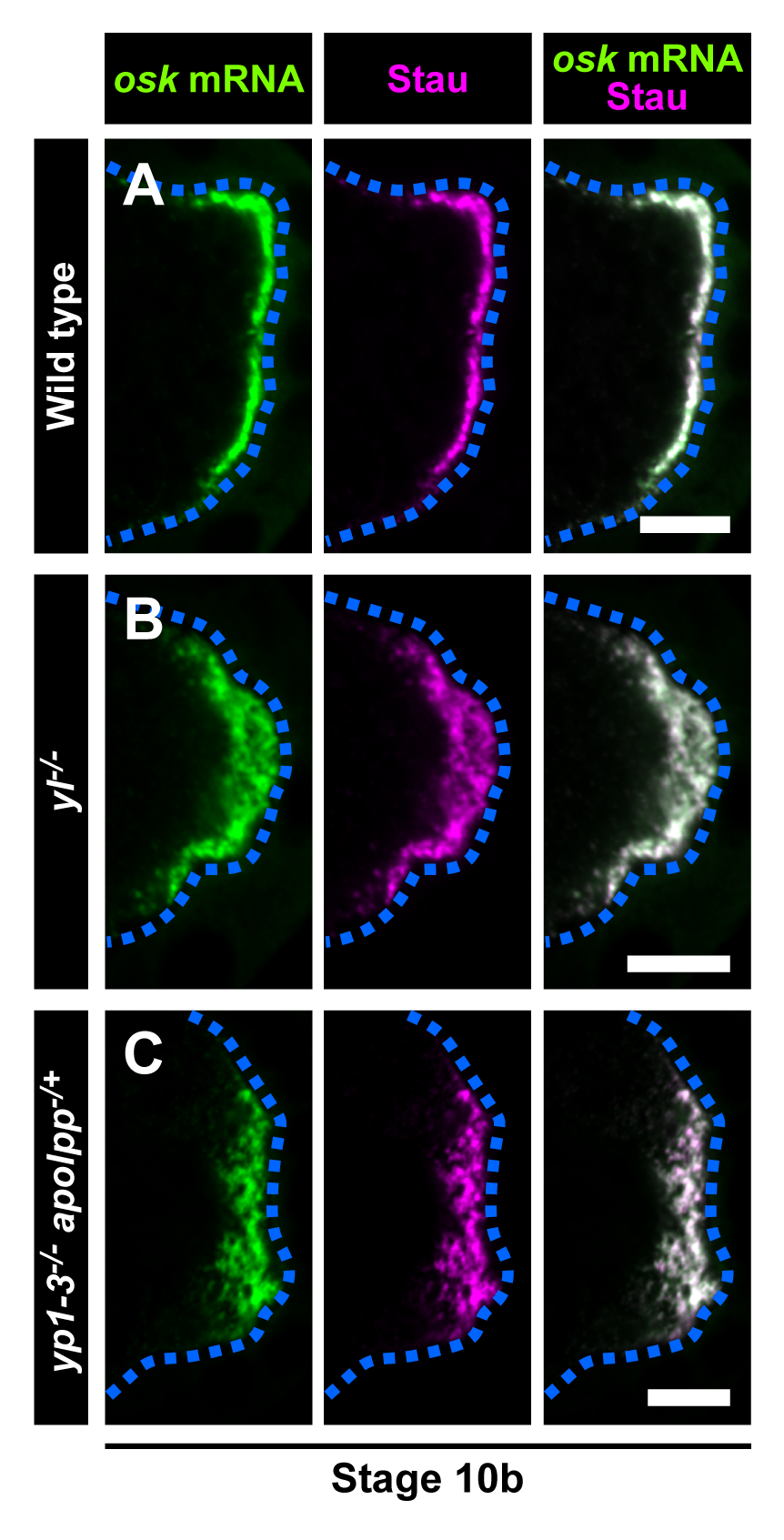

Supplement: S4 Fig — Posterior region of stage 10b oocytes stained for osk mRNA and Stau in wild-type (A), yl−/− (B), and yp1–3−/− apolpp−/+ (C) ovaries. Oocytes are outlined by blue dashed lines. Scale bars: 10 μm. Osk, Oskar; Stau, Staufen; Yl, Yolkless. (TIF) [file pbio.3001183.s004.tif]

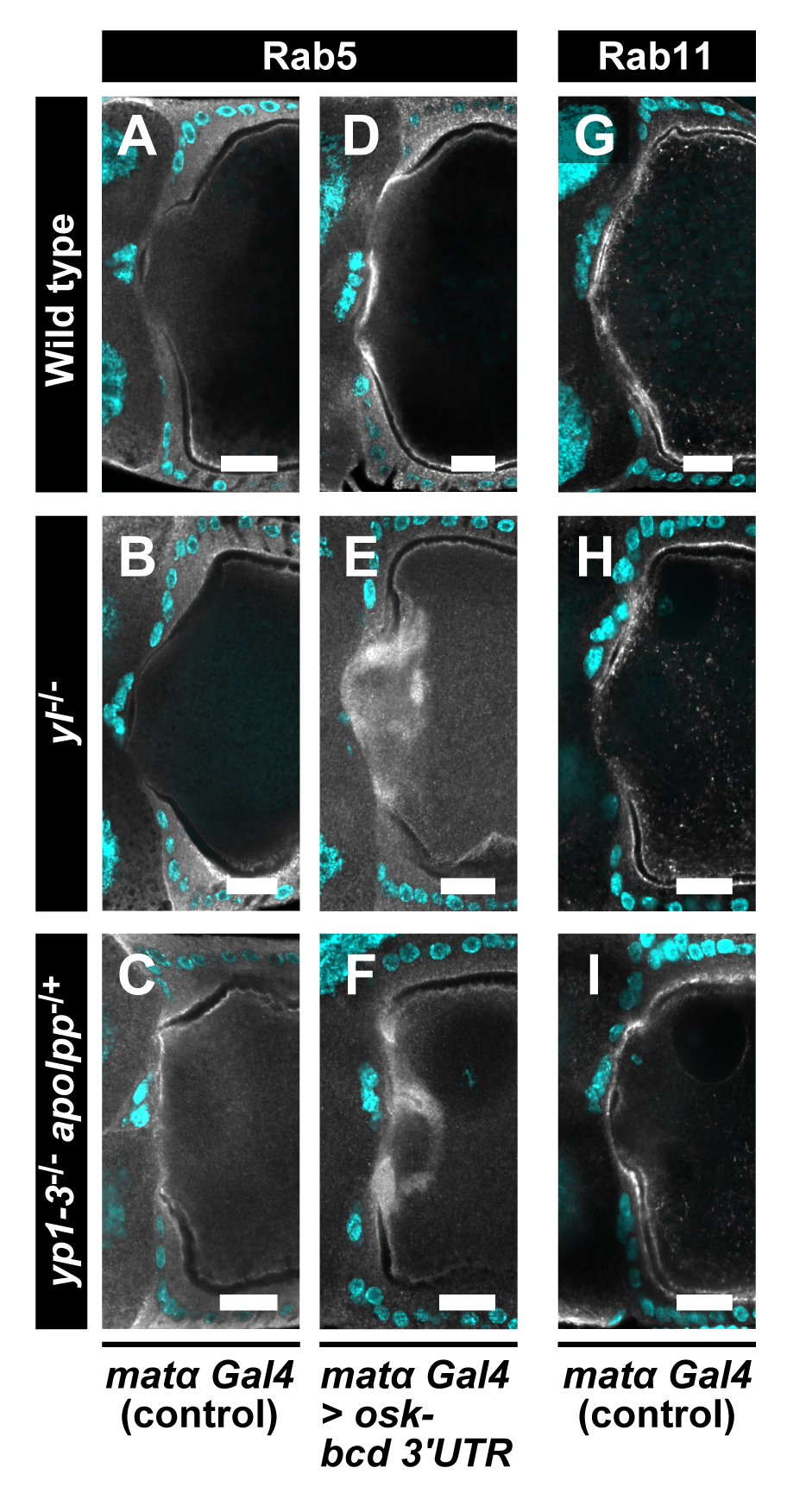

Supplement: S5 Fig — Rab5 (A-F) and Rab11 (G-I) at the anterior region of stage 10b oocytes without or with osk-bcd 3′UTR expression in wild-type (A, D, G), yl−/− (B, E, H), and yp1–3−/− apolpp−/+ (C, F, I) ovaries. Scale bars: 20 μm. Osk, Oskar; Yl, Yolkless. (TIF) [file pbio.3001183.s005.tif]

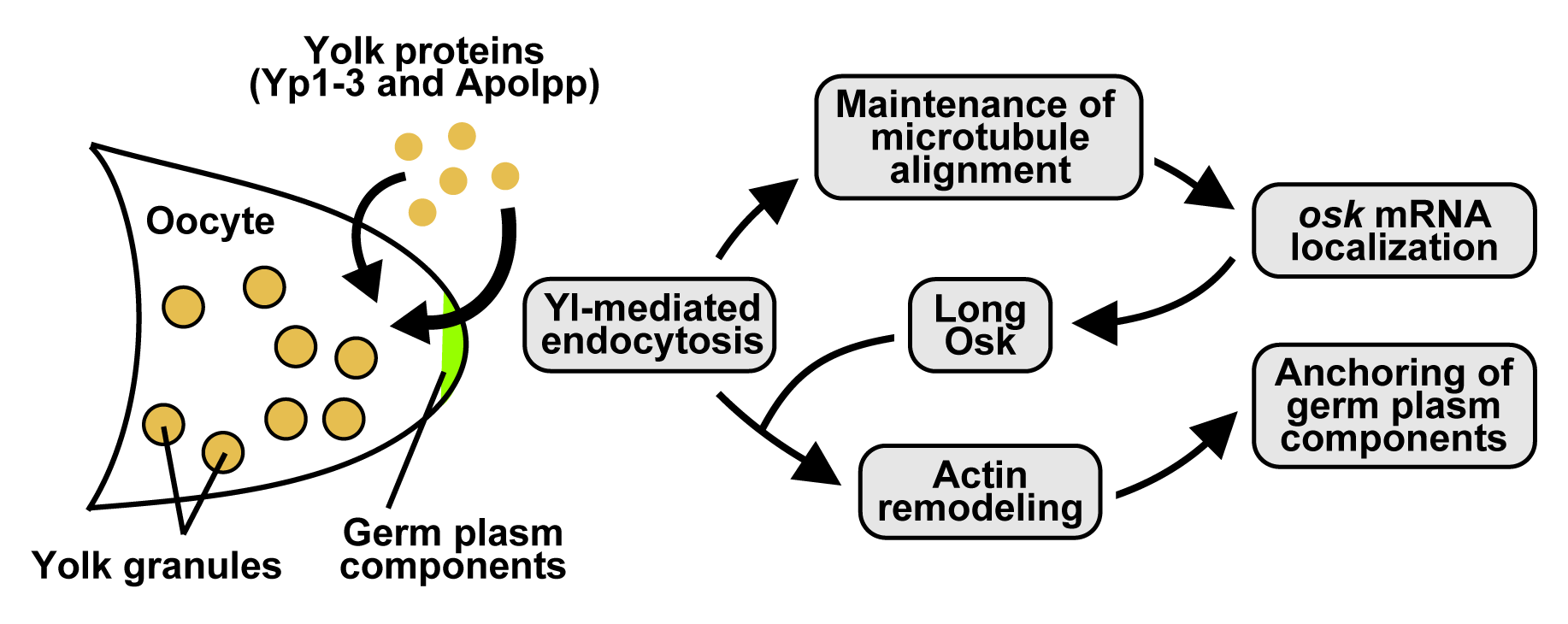

Supplement: S6 Fig — At vitellogenic stages, Yl binds to yolk proteins (Yp1–3 and Apolpp) at the oocyte surface. Ligand-bound Yl is then internalized by endocytosis and is delivered to endosomes. The yolk uptake process is required for maintenance of microtubule alignment to localize osk mRNA and long Osk-mediated actin remodeling to anchor germ plasm components to the oocyte posterior cortex. See the Discussion for detail. Apolpp, Apolipophorin; Osk, Oskar; Yl, Yolkless. (TIF) [file pbio.3001183.s006.tif]
