# Supplementary material for: Receptor-mediated yolk uptake is required for oskar mRNA localization and cortical anchorage of germ plasm components in the Drosophila oocyte
Source: PLoS Biol. 2021 Apr 23;19(4):e3001183. doi: 10.1371/journal.pbio.3001183 (PMC8064586; doi:10.1371/journal.pbio.3001183)
Supplement: S1 Table — (DOCX) [file pbio.3001183.s007.docx]

**S1 Table. A list of immunoprecipitated proteins with long and short Osk identified by mass spectrometry analysis**

| **L1** | | | | | | | | | | | |
| --- | --- | --- | --- | --- | --- | --- | --- | --- | --- | --- | --- |
| Family | Member | Database | Accession | Score | Mass | Num. of matches | Num. of significant matches | Num. of sequences | Num. of significant sequences | emPAI | Description |
| 1 | 1 | NCBInr | gi\|535346 | 1355 | 219176 | 120 | 75 | 72 | 51 | 1.95 | Yolkless |
| 2 | 1 | NCBInr | gi\|24642586 | 118 | 246515 | 19 | 9 | 19 | 9 | 0.14 | rudimentary, isoform A |
| 3 | 1 | NCBInr | gi\|2213915 | 110 | 127265 | 6 | 3 | 6 | 3 | 0.09 | cup |
| 4 | 1 | NCBInr | gi\|161076460 | 67 | 280962 | 13 | 5 | 13 | 5 | 0.07 | Not1, isoform C |
| 5 | 1 | NCBInr | gi\|28574898 | 67 | 244356 | 13 | 5 | 13 | 5 | 0.08 | lethal (3) 72Ab |
| 6 | 1 | NCBInr | gi\|281362724 | 65 | 178038 | 12 | 4 | 12 | 4 | 0.09 | La related protein, isoform D |
| 7 | 1 | NCBInr | gi\|24645205 | 55 | 69240 | 8 | 4 | 8 | 4 | 0.23 | oskar, isoform A |
| 8 | 1 | NCBInr | gi\|24647182 | 42 | 248838 | 7 | 2 | 7 | 2 | 0.03 | CG5205 |
| 9 | 1 | NCBInr | gi\|161076325 | 38 | 183829 | 9 | 2 | 9 | 2 | 0.04 | eukaryotic translation initiation factor 4G, isoform A |
| 10 | 1 | NCBInr | gi\|62862016 | 37 | 24310 | 1 | 1 | 1 | 1 | 0.16 | ribosomal protein L15, isoform A |
| 11 | 1 | NCBInr | gi\|157594 | 30 | 51979 | 4 | 3 | 4 | 3 | 0.23 | Me31B |
| 12 | 1 | NCBInr | gi\|158767 | 28 | 8540 | 2 | 2 | 2 | 2 | 1.18 | ubiquitin, partial |
| 13 | 1 | NCBInr | gi\|17975542 | 23 | 11374 | 1 | 1 | 1 | 1 | 0.35 | histone H4 replacement, isoform C |
| 14 | 1 | NCBInr | gi\|402747937 | 21 | 26361 | 2 | 1 | 1 | 1 | 0.14 | FBP2, partial |
| 15 | 1 | NCBInr | gi\|161077186 | 20 | 195871 | 4 | 1 | 2 | 1 | 0.02 | muscle wasted, isoform C |
| 16 | 1 | NCBInr | gi\|429892566 | 19 | 164336 | 1 | 1 | 1 | 1 | 0.02 | spindle E |
| 17 | 1 | NCBInr | gi\|24651924 | 17 | 34379 | 1 | 1 | 1 | 1 | 0.11 | CG8235 |
| 18 | 1 | NCBInr | gi\|992988 | 17 | 94896 | 1 | 1 | 1 | 1 | 0.04 | serotonin receptor 5-HT2 subtype |
| 19 | 1 | NCBInr | gi\|5911472 | 14 | 226791 | 1 | 1 | 1 | 1 | 0.02 | microtubule associated protein |
| 20 | 1 | NCBInr | gi\|20151637 | 14 | 106335 | 3 | 1 | 3 | 1 | 0.03 | abnormal spindle (asp) |
| 21 | 1 | NCBInr | gi\|19920632 | 14 | 266277 | 1 | 1 | 1 | 1 | 0.01 | CG3523, isoform A |
| **S1** | | | | | | | | | | | |
| Family | Member | Database | Accession | Score | Mass | Num. of matches | Num. of significant matches | Num. of sequences | Num. of significant sequences | emPAI | Description |
| 1 | 1 | NCBInr | gi\|24642586 | 329 | 246515 | 45 | 25 | 43 | 24 | 0.45 | rudimentary, isoform A |
| 2 | 1 | NCBInr | gi\|40882425 | 312 | 250169 | 61 | 27 | 56 | 27 | 0.48 | crinkled |
| 3 | 1 | NCBInr | gi\|535346 | 217 | 219176 | 34 | 17 | 32 | 17 | 0.33 | Yolkless |
| 4 | 1 | NCBInr | gi\|161076460 | 112 | 280962 | 13 | 8 | 13 | 8 | 0.11 | Not1, isoform C |
| 5 | 1 | NCBInr | gi\|281362724 | 95 | 178038 | 13 | 6 | 13 | 6 | 0.13 | La related protein, isoform D |
| 6 | 1 | NCBInr | gi\|28574898 | 88 | 244356 | 18 | 10 | 17 | 10 | 0.16 | lethal (3) 72Ab |
| 7 | 1 | NCBInr | gi\|2213915 | 86 | 127265 | 8 | 3 | 8 | 3 | 0.09 | cup |
| 8 | 1 | NCBInr | gi\|62862016 | 58 | 24310 | 1 | 1 | 1 | 1 | 0.16 | ribosomal protein L15, isoform A |
| 9 | 1 | NCBInr | gi\|161076325 | 41 | 183829 | 6 | 2 | 6 | 2 | 0.04 | eukaryotic translation initiation factor 4G, isoform A |
| 10 | 1 | NCBInr | gi\|157891 | 35 | 224288 | 8 | 2 | 8 | 2 | 0.03 | myosin heavy chain |
| 11 | 1 | NCBInr | gi\|24651924 | 25 | 34379 | 2 | 1 | 1 | 1 | 0.11 | CG8235 |
| 12 | 1 | NCBInr | gi\|156750 | 23 | 41797 | 1 | 1 | 1 | 1 | 0.09 | actin |
| 13 | 1 | NCBInr | gi\|402747937 | 21 | 26361 | 3 | 1 | 2 | 1 | 0.14 | FBP2, partial |
| 14 | 1 | NCBInr | gi\|41619560 | 20 | 23430 | 4 | 1 | 1 | 1 | 0.16 | TPA_inf: HDC14682 |
| 15 | 1 | NCBInr | gi\|19920632 | 17 | 266277 | 1 | 1 | 1 | 1 | 0.01 | CG3523, isoform A |
| 16 | 1 | NCBInr | gi\|24647182 | 16 | 248838 | 9 | 3 | 9 | 3 | 0.04 | CG5205 |
| 17 | 1 | NCBInr | gi\|24641865 | 16 | 46073 | 4 | 1 | 4 | 1 | 0.08 | yolk protein 3 |
| 18 | 1 | NCBInr | gi\|24645205 | 13 | 69240 | 1 | 1 | 1 | 1 | 0.05 | oskar, isoform A |
| **L2** | | | | | | | | | | | |
| Family | Member | Database | Accession | Score | Mass | Num. of matches | Num. of significant matches | Num. of sequences | Num. of significant sequences | emPAI | Description |
| 1 | 1 | NCBInr | gi\|2213915 | 1741 | 127265 | 129 | 88 | 53 | 45 | 7.52 | cup |
| 2 | 1 | NCBInr | gi\|24585795 | 418 | 143469 | 47 | 35 | 40 | 32 | 1.43 | CG17018, isoform A |
| 3 | 1 | NCBInr | gi\|24664664 | 229 | 136765 | 34 | 19 | 30 | 19 | 0.66 | argonaute 2, isoform B |
| 4 | 1 | NCBInr | gi\|24647162 | 111 | 117506 | 11 | 7 | 9 | 7 | 0.24 | Ataxin-2, isoform B |
| 5 | 1 | NCBInr | gi\|24645205 | 103 | 69240 | 9 | 7 | 8 | 6 | 0.44 | oskar, isoform A |
| 6 | 1 | NCBInr | gi\|71834239 | 82 | 125746 | 16 | 6 | 16 | 6 | 0.19 | eukaryotic translation initiation factor 3 subunit a |
| 7 | 1 | NCBInr | gi\|45549019 | 65 | 127701 | 8 | 4 | 8 | 4 | 0.12 | dre4, isoform A |
| 8 | 1 | NCBInr | gi\|158767 | 62 | 8540 | 3 | 3 | 3 | 3 | 2.23 | ubiquitin, partial |
| 9 | 1 | NCBInr | gi\|161076325 | 58 | 183829 | 8 | 2 | 8 | 2 | 0.04 | eukaryotic translation initiation factor 4G, isoform A |
| 10 | 1 | NCBInr | gi\|21355167 | 54 | 59706 | 5 | 3 | 5 | 3 | 0.2 | lost, isoform A |
| 11 | 1 | NCBInr | gi\|8214 | 53 | 143196 | 18 | 8 | 17 | 8 | 0.23 | myosin heavy chain |
| 12 | 1 | NCBInr | gi\|62862016 | 43 | 24310 | 4 | 2 | 4 | 2 | 0.34 | ribosomal protein L15, isoform A |
| 13 | 1 | NCBInr | gi\|442632449 | 35 | 110837 | 3 | 1 | 2 | 1 | 0.03 | Cep135, isoform C |
| 14 | 1 | NCBInr | gi\|2500374 | 27 | 16040 | 1 | 1 | 1 | 1 | 0.24 | Ribosomal protein L32 (RpL32) |
| 15 | 1 | NCBInr | gi\|24584738 | 25 | 121444 | 3 | 1 | 3 | 1 | 0.03 | CG31739, isoform A |
| 16 | 1 | NCBInr | gi\|157594 | 22 | 51979 | 6 | 2 | 5 | 2 | 0.15 | RNA helicase |
| 17 | 1 | NCBInr | gi\|402747937 | 21 | 26361 | 2 | 1 | 2 | 1 | 0.14 | FBP2, partial |
| 18 | 1 | NCBInr | gi\|24651203 | 21 | 77944 | 1 | 1 | 1 | 1 | 0.05 | CG31038, isoform A |
| 19 | 1 | NCBInr | gi\|397852 | 20 | 17123 | 1 | 1 | 1 | 1 | 0.23 | ribosomal protein L27a |
| 20 | 1 | NCBInr | gi\|41619560 | 20 | 23430 | 4 | 1 | 1 | 1 | 0.16 | TPA_inf: HDC14682 |
| 21 | 1 | NCBInr | gi\|28316833 | 19 | 137707 | 5 | 1 | 5 | 1 | 0.03 | rigor mortis (rig) |
| 22 | 1 | NCBInr | gi\|24651924 | 18 | 34379 | 1 | 1 | 1 | 1 | 0.11 | CG8235 |
| 23 | 1 | NCBInr | gi\|535346 | 18 | 219176 | 1 | 1 | 1 | 1 | 0.02 | vitellogenin receptor |
| 24 | 1 | NCBInr | gi\|8188 | 17 | 108434 | 3 | 1 | 3 | 1 | 0.03 | lodestar protein |
| 25 | 1 | NCBInr | gi\|21357777 | 16 | 32346 | 1 | 1 | 1 | 1 | 0.12 | CG6833, isoform A |
| 26 | 1 | NCBInr | gi\|48596237 | 16 | 103951 | 1 | 1 | 1 | 1 | 0.04 | gag protein |
| 27 | 1 | NCBInr | gi\|19920632 | 15 | 266277 | 1 | 1 | 1 | 1 | 0.01 | CG3523, isoform A |
| 28 | 1 | NCBInr | gi\|45550607 | 15 | 69298 | 1 | 1 | 1 | 1 | 0.05 | trailer hitch, isoform A |
| 29 | 1 | NCBInr | gi\|21358185 | 14 | 155215 | 1 | 1 | 1 | 1 | 0.02 | CG11897, isoform B |
| **S2** | | | | | | | | | | | |
| Family | Member | Database | Accession | Score | Mass | Num. of matches | Num. of significant matches | Num. of sequences | Num. of significant sequences | emPAI | Description |
| 1 | 1 | NCBInr | gi\|2213915 | 2642 | 127265 | 178 | 118 | 54 | 46 | 12.07 | cup |
| 2 | 2 | NCBInr | gi\|221473199 | 2618 | 125594 | 177 | 117 | 54 | 46 | 12.17 | cup |
| 3 | 1 | NCBInr | gi\|80979033 | 575 | 95704 | 45 | 31 | 39 | 28 | 2.12 | Argonaute-2 |
| 4 | 2 | NCBInr | gi\|80979035 | 567 | 95717 | 45 | 31 | 39 | 28 | 2.12 | Argonaute-2 |
| 5 | 1 | NCBInr | gi\|24585795 | 551 | 143469 | 43 | 28 | 38 | 25 | 0.93 | CG17018, isoform A |
| 6 | 1 | NCBInr | gi\|157594 | 195 | 51979 | 19 | 12 | 17 | 11 | 1.3 | Me31B |
| 7 | 1 | NCBInr | gi\|24643988 | 188 | 133798 | 38 | 16 | 36 | 14 | 0.5 | eIF3-S10 |
| 8 | 1 | NCBInr | gi\|28316833 | 158 | 137707 | 21 | 10 | 21 | 10 | 0.3 | rigor mortis (rig) |
| 9 | 1 | NCBInr | gi\|17137608 | 151 | 139241 | 16 | 9 | 15 | 9 | 0.27 | alpha-coatomer protein, isoform A |
| 10 | 1 | NCBInr | gi\|45549019 | 132 | 127701 | 19 | 7 | 19 | 7 | 0.22 | dre4, isoform A |
| 11 | 1 | NCBInr | gi\|24647162 | 112 | 117506 | 15 | 8 | 11 | 7 | 0.28 | Ataxin-2, isoform B |
| 12 | 1 | NCBInr | gi\|157906 | 111 | 143498 | 20 | 7 | 18 | 7 | 0.19 | maleless protein |
| 13 | 1 | NCBInr | gi\|45550087 | 101 | 149543 | 11 | 5 | 11 | 5 | 0.13 | CG2807, isoform A |
| 14 | 1 | NCBInr | gi\|24584738 | 93 | 121444 | 11 | 5 | 11 | 5 | 0.16 | CG31739, isoform A |
| 15 | 1 | NCBInr | gi\|45553317 | 73 | 53644 | 5 | 3 | 5 | 3 | 0.22 | oskar, isoform C |
| 16 | 1 | NCBInr | gi\|47156225 | 73 | 135611 | 8 | 5 | 8 | 5 | 0.14 | armitage |
| 17 | 1 | NCBInr | gi\|161076325 | 56 | 183829 | 16 | 5 | 16 | 5 | 0.1 | eukaryotic translation initiation factor 4G, isoform A |
| 18 | 1 | NCBInr | gi\|21711689 | 56 | 143187 | 10 | 5 | 10 | 5 | 0.14 | CG6701 |
| 19 | 1 | NCBInr | gi\|62862016 | 50 | 24310 | 4 | 2 | 4 | 2 | 0.34 | ribosomal protein L15, isoform A |
| 20 | 1 | NCBInr | gi\|21355167 | 47 | 59706 | 6 | 3 | 6 | 3 | 0.2 | lost, isoform A |
| 21 | 1 | NCBInr | gi\|24583646 | 44 | 160174 | 3 | 1 | 3 | 1 | 0.02 | nucleoporin 160 |
| 22 | 1 | NCBInr | gi\|158769 | 39 | 8428 | 3 | 2 | 3 | 2 | 1.21 | ubiquitin, partial |
| 23 | 1 | NCBInr | gi\|45550607 | 38 | 69298 | 8 | 1 | 8 | 1 | 0.05 | trailer hitch, isoform A |
| 24 | 1 | NCBInr | gi\|24640488 | 29 | 139968 | 8 | 2 | 8 | 2 | 0.05 | Upf2 |
| 25 | 1 | NCBInr | gi\|8488 | 28 | 45727 | 2 | 1 | 2 | 1 | 0.08 | Ribosomal protein L4 (RpL4) |
| 26 | 1 | NCBInr | gi\|17737290 | 27 | 28390 | 1 | 1 | 1 | 1 | 0.13 | ribosomal protein S6, isoform B |
| 27 | 1 | NCBInr | gi\|24653655 | 27 | 160617 | 2 | 1 | 2 | 1 | 0.02 | cleavage and polyadenylation specificity factor 160, isoform B |
| 28 | 1 | NCBInr | gi\|19920632 | 25 | 266277 | 1 | 1 | 1 | 1 | 0.01 | CG3523, isoform A |
| 29 | 1 | NCBInr | gi\|41619560 | 20 | 23430 | 3 | 1 | 1 | 1 | 0.16 | TPA_inf: HDC14682 |
| 30 | 1 | NCBInr | gi\|17136376 | 19 | 91095 | 1 | 1 | 1 | 1 | 0.04 | armadillo, isoform A |
| 31 | 1 | NCBInr | gi\|297372440 | 18 | 32952 | 1 | 1 | 1 | 1 | 0.12 | gustatory receptor 93c |
| 32 | 1 | NCBInr | gi\|21392184 | 17 | 93705 | 5 | 2 | 5 | 2 | 0.08 | lodestar (lds) |
| 33 | 1 | NCBInr | gi\|28573062 | 16 | 25600 | 1 | 1 | 1 | 1 | 0.15 | CG10005, isoform A |
| 34 | 1 | NCBInr | gi\|156750 | 14 | 41797 | 2 | 1 | 2 | 1 | 0.09 | actin |
| 35 | 1 | NCBInr | gi\|429892566 | 14 | 164336 | 1 | 1 | 1 | 1 | 0.02 | spindle E |
| 36 | 1 | NCBInr | gi\|7025386 | 14 | 150831 | 7 | 1 | 7 | 1 | 0.02 | La related protein |
| **L3** | | | | | | | | | | | |
| Family | Member | Database | Accession | Score | Mass | Num. of matches | Num. of significant matches | Num. of sequences | Num. of significant sequences | emPAI | Description |
| 1 | 1 | NCBInr | gi\|24645205 | 1176 | 69240 | 82 | 55 | 36 | 28 | 10.64 | oskar, isoform A |
| 2 | 1 | NCBInr | gi\|7919 | 374 | 94412 | 36 | 23 | 32 | 22 | 1.42 | eukaryotic translation elongation factor 2 (eEF2) |
| 3 | 1 | NCBInr | gi\|17136720 | 253 | 97788 | 17 | 14 | 16 | 14 | 0.68 | bicaudal C, isoform A |
| 4 | 1 | NCBInr | gi\|21356859 | 214 | 102212 | 21 | 13 | 17 | 12 | 0.59 | regulatory particle non-ATPase 1 |
| 5 | 1 | NCBInr | gi\|429892788 | 122 | 98441 | 24 | 10 | 22 | 9 | 0.45 | aubergine |
| 6 | 1 | NCBInr | gi\|18857967 | 111 | 100356 | 12 | 8 | 12 | 8 | 0.34 | CG10777 |
| 7 | 1 | NCBInr | gi\|8307 | 110 | 98660 | 16 | 7 | 12 | 6 | 0.29 | oo18 RNA-binding protein (Orb) |
| 8 | 1 | NCBInr | gi\|45553181 | 103 | 75553 | 13 | 6 | 12 | 6 | 0.33 | protein on ecdysone puffs, isoform C |
| 9 | 1 | NCBInr | gi\|2213915 | 99 | 127265 | 7 | 4 | 7 | 4 | 0.12 | cup |
| 10 | 1 | NCBInr | gi\|21355167 | 85 | 59706 | 12 | 7 | 10 | 6 | 0.53 | lost, isoform A |
| 11 | 1 | NCBInr | gi\|4235475 | 61 | 96942 | 7 | 4 | 7 | 4 | 0.16 | topoisomerase III |
| 12 | 1 | NCBInr | gi\|158029 | 60 | 92553 | 7 | 5 | 7 | 5 | 0.22 | ovarian tumor protein isoform |
| 13 | 1 | NCBInr | gi\|62862016 | 55 | 24310 | 3 | 1 | 3 | 1 | 0.16 | ribosomal protein L15, isoform A |
| 14 | 1 | NCBInr | gi\|20130109 | 49 | 86539 | 5 | 3 | 4 | 3 | 0.13 | CG5726 |
| 15 | 1 | NCBInr | gi\|17737290 | 37 | 28390 | 1 | 1 | 1 | 1 | 0.13 | ribosomal protein S6, isoform B |
| 16 | 1 | NCBInr | gi\|158759 | 32 | 8548 | 1 | 1 | 1 | 1 | 0.48 | ubiquitin, partial |
| 17 | 1 | NCBInr | gi\|3204159 | 29 | 102633 | 2 | 1 | 2 | 1 | 0.04 | coatomer, beta-prime subunit |
| 18 | 1 | NCBInr | gi\|21429090 | 28 | 93265 | 3 | 1 | 3 | 1 | 0.04 | CG6967 |
| 19 | 1 | NCBInr | gi\|7831 | 27 | 93700 | 2 | 1 | 2 | 1 | 0.04 | dynamin |
| 20 | 1 | NCBInr | gi\|17647879 | 24 | 24936 | 2 | 1 | 2 | 1 | 0.15 | ribosomal protein L13, isoform A |
| 21 | 1 | NCBInr | gi\|429892566 | 24 | 164336 | 1 | 1 | 1 | 1 | 0.02 | spindle E |
| 22 | 1 | NCBInr | gi\|19921596 | 21 | 89849 | 1 | 1 | 1 | 1 | 0.04 | centrocortin, isoform A |
| 23 | 1 | NCBInr | gi\|10242347 | 20 | 85037 | 1 | 1 | 1 | 1 | 0.04 | SR protein kinase 1 |
| 24 | 1 | NCBInr | gi\|24668014 | 20 | 93658 | 2 | 1 | 2 | 1 | 0.04 | CG7338 |
| 25 | 1 | NCBInr | gi\|17647193 | 18 | 107339 | 3 | 1 | 3 | 1 | 0.03 | beta-coatomer protein |
| 26 | 1 | NCBInr | gi\|19920632 | 18 | 266277 | 1 | 1 | 1 | 1 | 0.01 | CG3523, isoform A |
| 27 | 1 | NCBInr | gi\|8488 | 17 | 45727 | 3 | 1 | 3 | 1 | 0.08 | Ribosomal protein L4 (RpL4) |
| 28 | 1 | NCBInr | gi\|402747937 | 17 | 26361 | 2 | 1 | 2 | 1 | 0.14 | FBP2, partial |
| **S3** | | | | | | | | | | | |
| Family | Member | Database | Accession | Score | Mass | Num. of matches | Num. of significant matches | Num. of sequences | Num. of significant sequences | emPAI | Description |
| 1 | 1 | NCBInr | gi\|17136720 | 468 | 97788 | 50 | 33 | 30 | 21 | 1.72 | bicaudal C, isoform A |
| 2 | 1 | NCBInr | gi\|2213915 | 175 | 127265 | 25 | 14 | 21 | 13 | 0.49 | cup |
| 3 | 1 | NCBInr | gi\|24584107 | 143 | 102648 | 26 | 13 | 25 | 12 | 0.58 | beta'-coatomer protein |
| 4 | 1 | NCBInr | gi\|8307 | 137 | 98660 | 11 | 7 | 9 | 7 | 0.29 | ovarian protein |
| 5 | 1 | NCBInr | gi\|24642763 | 127 | 109344 | 16 | 8 | 16 | 8 | 0.3 | CG8915 |
| 6 | 1 | NCBInr | gi\|28574893 | 115 | 95968 | 14 | 6 | 13 | 6 | 0.25 | Zinc-finger protein at 72D, isoform B |
| 7 | 1 | NCBInr | gi\|21358245 | 112 | 91672 | 15 | 8 | 14 | 7 | 0.37 | CG13472 |
| 8 | 1 | NCBInr | gi\|24583962 | 90 | 100197 | 22 | 12 | 18 | 10 | 0.54 | CG5787, isoform A |
| 9 | 2 | NCBInr | gi\|18857967 | 53 | 100356 | 7 | 3 | 7 | 3 | 0.11 | CG10777 |
| 10 | 1 | NCBInr | gi\|157594 | 65 | 51979 | 9 | 4 | 9 | 4 | 0.32 | RNA helicase |
| 11 | 1 | NCBInr | gi\|62862016 | 53 | 24310 | 2 | 1 | 2 | 1 | 0.16 | ribosomal protein L15, isoform A |
| 12 | 1 | NCBInr | gi\|21357605 | 41 | 105665 | 4 | 2 | 4 | 2 | 0.07 | CG14476, isoform B |
| 13 | 1 | NCBInr | gi\|24643243 | 40 | 103027 | 11 | 4 | 11 | 4 | 0.15 | Nat1 |
| 14 | 1 | NCBInr | gi\|17647193 | 36 | 107339 | 5 | 3 | 5 | 3 | 0.11 | beta-coatomer protein |
| 15 | 1 | NCBInr | gi\|21355167 | 34 | 59706 | 5 | 2 | 5 | 2 | 0.13 | lost, isoform A |
| 16 | 1 | NCBInr | gi\|16768302 | 33 | 73015 | 5 | 1 | 5 | 1 | 0.05 | GM01081p |
| 17 | 1 | NCBInr | gi\|45553317 | 32 | 53644 | 3 | 2 | 3 | 2 | 0.14 | oskar, isoform C |
| 18 | 1 | NCBInr | gi\|8488 | 27 | 45727 | 1 | 1 | 1 | 1 | 0.08 | Ribosomal protein L4 (RpL4) |
| 19 | 1 | NCBInr | gi\|3928792 | 22 | 91967 | 2 | 1 | 2 | 1 | 0.04 | SIR2 |
| 20 | 1 | NCBInr | gi\|25012282 | 22 | 117979 | 1 | 1 | 1 | 1 | 0.03 | CG3744 |
| 21 | 1 | NCBInr | gi\|8484 | 20 | 28188 | 1 | 1 | 1 | 1 | 0.13 | Ribosomal protein L7 (RpL7) |
| 22 | 1 | NCBInr | gi\|41619560 | 20 | 23430 | 3 | 1 | 1 | 1 | 0.16 | TPA_inf: HDC14682 |
| 23 | 1 | NCBInr | gi\|17864514 | 19 | 78000 | 3 | 1 | 2 | 1 | 0.05 | protein on ecdysone puffs, isoform B |
| 24 | 1 | NCBInr | gi\|158767 | 19 | 8540 | 2 | 2 | 2 | 2 | 1.18 | ubiquitin, partial |
| 25 | 1 | NCBInr | gi\|675456 | 19 | 102720 | 2 | 2 | 2 | 2 | 0.07 | Spellchecker1, partial |
| 26 | 1 | NCBInr | gi\|429892566 | 17 | 164336 | 1 | 1 | 1 | 1 | 0.02 | spindle E |
| 27 | 1 | NCBInr | gi\|402747937 | 17 | 26361 | 1 | 1 | 1 | 1 | 0.14 | FBP2, partial |
| 28 | 1 | NCBInr | gi\|13124689 | 17 | 106952 | 5 | 1 | 5 | 1 | 0.03 | Alpha-actinin |
| 29 | 1 | NCBInr | gi\|28573062 | 16 | 25600 | 1 | 1 | 1 | 1 | 0.15 | CG10005, isoform A |
| 30 | 1 | NCBInr | gi\|17647145 | 16 | 106150 | 4 | 1 | 4 | 1 | 0.03 | Argonaute-1, isoform B |
| 31 | 1 | NCBInr | gi\|28558744 | 15 | 113820 | 3 | 1 | 3 | 1 | 0.03 | CG32016, isoform B |
| 32 | 1 | NCBInr | gi\|21356859 | 13 | 102212 | 1 | 1 | 1 | 1 | 0.04 | Regulatory particle non-ATPase 1 (Rpn1) |
| **L4** | | | | | | | | | | | |
| Family | Member | Database | Accession | Score | Mass | Num. of matches | Num. of significant matches | Num. of sequences | Num. of significant sequences | emPAI | Description |
| 1 | 1 | NCBInr | gi\|24645205 | 441 | 69240 | 34 | 23 | 23 | 18 | 2.32 | oskar, isoform A |
| 2 | 1 | NCBInr | gi\|17647529 | 356 | 81814 | 29 | 17 | 26 | 16 | 1.03 | heat shock protein 83, isoform A |
| 3 | 1 | NCBInr | gi\|28574998 | 283 | 72576 | 22 | 16 | 17 | 12 | 1.11 | cueball |
| 4 | 1 | NCBInr | gi\|19922726 | 241 | 76030 | 23 | 15 | 18 | 14 | 1.04 | Fmr1, isoform A |
| 5 | 1 | NCBInr | gi\|7662 | 237 | 77005 | 20 | 17 | 19 | 16 | 1.12 | Bj6 protein |
| 6 | 1 | NCBInr | gi\|21355167 | 150 | 59706 | 17 | 9 | 13 | 8 | 0.72 | lost, isoform A |
| 7 | 1 | NCBInr | gi\|7739653 | 132 | 74940 | 17 | 7 | 13 | 7 | 0.4 | rasputin |
| 8 | 1 | NCBInr | gi\|17985987 | 75 | 85029 | 4 | 2 | 4 | 2 | 0.09 | belle, isoform A |
| 9 | 1 | NCBInr | gi\|24646386 | 68 | 84572 | 12 | 7 | 11 | 7 | 0.35 | malic enzyme, isoform B |
| 10 | 1 | NCBInr | gi\|442631973 | 65 | 69711 | 9 | 4 | 8 | 4 | 0.23 | trailer hitch, isoform C |
| 11 | 1 | NCBInr | gi\|24640411 | 63 | 137356 | 7 | 4 | 7 | 4 | 0.11 | defective chorion 1, isoform B |
| 12 | 1 | NCBInr | gi\|3953611 | 62 | 81003 | 3 | 2 | 3 | 2 | 0.09 | MCM7 |
| 13 | 1 | NCBInr | gi\|28574962 | 55 | 88155 | 3 | 1 | 2 | 1 | 0.04 | CG10077, isoform A |
| 14 | 1 | NCBInr | gi\|157594 | 46 | 51979 | 4 | 3 | 4 | 3 | 0.23 | Me31B |
| 15 | 1 | NCBInr | gi\|17737290 | 40 | 28390 | 2 | 2 | 2 | 2 | 0.28 | ribosomal protein S6, isoform B |
| 16 | 1 | NCBInr | gi\|24644436 | 39 | 80709 | 4 | 1 | 4 | 1 | 0.05 | Hpr1 |
| 17 | 1 | NCBInr | gi\|62862016 | 39 | 24310 | 3 | 1 | 3 | 1 | 0.16 | ribosomal protein L15, isoform A |
| 18 | 1 | NCBInr | gi\|4378006 | 35 | 28609 | 1 | 1 | 1 | 1 | 0.13 | ribosomal protein L23a |
| 19 | 1 | NCBInr | gi\|116007410 | 35 | 77508 | 3 | 1 | 3 | 1 | 0.05 | CG40006 |
| 20 | 1 | NCBInr | gi\|24583077 | 28 | 79588 | 5 | 2 | 5 | 2 | 0.1 | mitochondrial trifunctional protein alpha subunit, isoform B |
| 21 | 1 | NCBInr | gi\|24583248 | 27 | 29534 | 3 | 2 | 3 | 2 | 0.27 | ribosomal protein L7 |
| 22 | 1 | NCBInr | gi\|7735 | 27 | 77453 | 4 | 1 | 4 | 1 | 0.05 | claret segregational product, claret disjunctin |
| 23 | 1 | NCBInr | gi\|21356169 | 25 | 78947 | 2 | 2 | 2 | 2 | 0.1 | Nnp-1 |
| 24 | 1 | NCBInr | gi\|24651924 | 25 | 34379 | 2 | 1 | 1 | 1 | 0.11 | CG8235 |
| 25 | 1 | NCBInr | gi\|21357161 | 24 | 29718 | 2 | 1 | 2 | 1 | 0.13 | ribosomal protein L6, isoform B |
| 26 | 1 | NCBInr | gi\|158767 | 23 | 8540 | 3 | 1 | 3 | 1 | 0.48 | ubiquitin, partial |
| 27 | 1 | NCBInr | gi\|24581168 | 21 | 82515 | 3 | 2 | 3 | 2 | 0.09 | dynamin related protein 1, isoform A |
| 28 | 1 | NCBInr | gi\|41619560 | 20 | 23430 | 3 | 1 | 1 | 1 | 0.16 | TPA_inf: HDC14682 |
| 29 | 1 | NCBInr | gi\|402747937 | 19 | 26361 | 2 | 2 | 1 | 1 | 0.14 | FBP2, partial |
| 30 | 1 | NCBInr | gi\|19920632 | 15 | 266277 | 1 | 1 | 1 | 1 | 0.01 | CG3523, isoform A |
| 31 | 1 | NCBInr | gi\|19922458 | 15 | 80391 | 4 | 1 | 4 | 1 | 0.05 | eIF3-S9, isoform B |
| 32 | 1 | NCBInr | gi\|158189 | 14 | 167306 | 1 | 1 | 1 | 1 | 0.02 | Protein tyrosine phosphatase 69D (Ptp69D) |
| **S4** | | | | | | | | | | | |
| Family | Member | Database | Accession | Score | Mass | Num. of matches | Num. of significant matches | Num. of sequences | Num. of significant sequences | emPAI | Description |
| 1 | 1 | NCBInr | gi\|45553317 | 2022 | 53644 | 123 | 92 | 28 | 25 | 63.62 | oskar, isoform C |
| 2 | 1 | NCBInr | gi\|17647529 | 561 | 81814 | 35 | 28 | 28 | 24 | 2.31 | heat shock protein 83, isoform A |
| 3 | 1 | NCBInr | gi\|442631973 | 534 | 69711 | 35 | 23 | 22 | 17 | 1.97 | trailer hitch, isoform C |
| 4 | 1 | NCBInr | gi\|24645595 | 502 | 75773 | 38 | 25 | 23 | 19 | 2 | Fmr1, isoform C |
| 5 | 1 | NCBInr | gi\|7662 | 288 | 77005 | 19 | 13 | 18 | 13 | 0.84 | Bj6 protein |
| 6 | 1 | NCBInr | gi\|19922458 | 217 | 80391 | 15 | 11 | 15 | 11 | 0.64 | eIF3-S9, isoform B |
| 7 | 1 | NCBInr | gi\|21355167 | 201 | 59706 | 25 | 14 | 18 | 12 | 1.33 | lost, isoform A |
| 8 | 1 | NCBInr | gi\|290280 | 198 | 81645 | 15 | 12 | 14 | 12 | 0.7 | single-stranded recognition protein |
| 9 | 1 | NCBInr | gi\|19921728 | 184 | 82599 | 18 | 9 | 18 | 9 | 0.48 | CG11107, isoform A |
| 10 | 1 | NCBInr | gi\|17985987 | 180 | 85029 | 19 | 11 | 18 | 10 | 0.6 | belle, isoform A |
| 11 | 1 | NCBInr | gi\|24646611 | 177 | 74898 | 19 | 10 | 16 | 10 | 0.62 | rasputin, isoform B |
| 12 | 1 | NCBInr | gi\|17647617 | 166 | 81232 | 15 | 8 | 13 | 8 | 0.43 | minichromosome maintenance 7 |
| 13 | 1 | NCBInr | gi\|17136720 | 111 | 97788 | 8 | 5 | 7 | 5 | 0.2 | bicaudal C, isoform A |
| 14 | 1 | NCBInr | gi\|2213915 | 108 | 127265 | 5 | 4 | 5 | 4 | 0.12 | cup |
| 15 | 1 | NCBInr | gi\|24640411 | 86 | 137356 | 11 | 4 | 11 | 4 | 0.11 | defective chorion 1, isoform B |
| 16 | 1 | NCBInr | gi\|24583077 | 77 | 79588 | 8 | 3 | 7 | 3 | 0.15 | mitochondrial trifunctional protein alpha subunit, isoform B |
| 17 | 1 | NCBInr | gi\|158767 | 66 | 8540 | 3 | 3 | 3 | 3 | 2.23 | ubiquitin, partial |
| 18 | 1 | NCBInr | gi\|17136376 | 50 | 91095 | 2 | 1 | 2 | 1 | 0.04 | armadillo, isoform A |
| 19 | 1 | NCBInr | gi\|3869123 | 49 | 80634 | 6 | 2 | 6 | 2 | 0.09 | DREF transcription factor |
| 20 | 1 | NCBInr | gi\|20130109 | 46 | 86539 | 4 | 2 | 4 | 2 | 0.09 | CG5726 |
| 21 | 1 | NCBInr | gi\|62862016 | 41 | 24310 | 4 | 2 | 4 | 2 | 0.34 | ribosomal protein L15, isoform A |
| 22 | 1 | NCBInr | gi\|7735 | 40 | 77453 | 7 | 2 | 6 | 2 | 0.1 | claret segregational product, claret disjunctin |
| 23 | 1 | NCBInr | gi\|24644436 | 40 | 80709 | 5 | 3 | 5 | 3 | 0.14 | Hpr1 |
| 24 | 1 | NCBInr | gi\|24581168 | 36 | 82515 | 6 | 2 | 5 | 2 | 0.09 | dynamin related protein 1, isoform A |
| 25 | 1 | NCBInr | gi\|6746602 | 36 | 67297 | 2 | 2 | 2 | 2 | 0.11 | La related protein 7 (Larp7) |
| 26 | 1 | NCBInr | gi\|21357161 | 31 | 29718 | 1 | 1 | 1 | 1 | 0.13 | ribosomal protein L6, isoform B |
| 27 | 1 | NCBInr | gi\|17137126 | 23 | 84209 | 2 | 2 | 2 | 2 | 0.09 | crooked neck |
| 28 | 1 | NCBInr | gi\|24585440 | 26 | 77515 | 2 | 1 | 2 | 1 | 0.05 | cdc23, isoform A |
| 29 | 1 | NCBInr | gi\|78214297 | 25 | 103844 | 2 | 1 | 2 | 1 | 0.04 | CG17258 |
| 30 | 1 | NCBInr | gi\|17530825 | 21 | 30713 | 6 | 2 | 5 | 2 | 0.26 | ribosomal protein L7A, isoform D |
| 31 | 1 | NCBInr | gi\|24665814 | 22 | 76019 | 3 | 1 | 3 | 1 | 0.05 | CG6512, isoform B |
| 32 | 1 | NCBInr | gi\|6946680 | 21 | 82201 | 2 | 1 | 2 | 1 | 0.05 | Zeste-white 10 (Zw10) |
| 33 | 1 | NCBInr | gi\|24651924 | 21 | 34379 | 2 | 1 | 1 | 1 | 0.11 | CG8235 |
| 34 | 1 | NCBInr | gi\|41619560 | 20 | 23430 | 4 | 1 | 1 | 1 | 0.16 | TPA_inf: HDC14682 |
| 35 | 1 | NCBInr | gi\|402747937 | 19 | 26361 | 3 | 1 | 2 | 1 | 0.14 | FBP2, partial |
| 36 | 1 | NCBInr | gi\|21686989 | 19 | 78797 | 1 | 1 | 1 | 1 | 0.05 | CG12942 |
| 37 | 1 | NCBInr | gi\|419961 | 18 | 65846 | 2 | 1 | 2 | 1 | 0.06 | poly(A) binding protein (pAbp) |
| 38 | 1 | NCBInr | gi\|17737290 | 18 | 28390 | 3 | 1 | 3 | 1 | 0.13 | ribosomal protein S6, isoform B |
| 39 | 1 | NCBInr | gi\|17647879 | 17 | 24936 | 1 | 1 | 1 | 1 | 0.15 | ribosomal protein L13, isoform A |
| 40 | 1 | NCBInr | gi\|157594 | 17 | 51979 | 5 | 2 | 5 | 2 | 0.15 | Me31B |
| 41 | 1 | NCBInr | gi\|8488 | 16 | 45727 | 2 | 1 | 2 | 1 | 0.08 | Ribosomal protein L4 (RpL4) |
| **L5** | | | | | | | | | | | |
| Family | Member | Database | Accession | Score | Mass | Num. of matches | Num. of significant matches | Num. of sequences | Num. of significant sequences | emPAI | Description |
| 1 | 1 | NCBInr | gi\|442631979 | 2929 | 69385 | 149 | 108 | 38 | 32 | 54.4 | trailer hitch, isoform F |
| 2 | 2 | NCBInr | gi\|442631975 | 2807 | 68675 | 141 | 100 | 35 | 30 | 36.79 | trailer hitch, isoform D |
| 3 | 1 | NCBInr | gi\|21355167 | 397 | 59706 | 32 | 26 | 23 | 19 | 3.27 | lost, isoform A |
| 4 | 1 | NCBInr | gi\|1511646 | 234 | 372414 | 20 | 10 | 18 | 9 | 0.1 | Apolipophorin |
| 5 | 1 | NCBInr | gi\|24645205 | 93 | 69240 | 10 | 7 | 9 | 6 | 0.44 | oskar, isoform A |
| 6 | 1 | NCBInr | gi\|157665 | 60 | 71015 | 11 | 6 | 10 | 5 | 0.36 | heat shock cognate 4 |
| 7 | 1 | NCBInr | gi\|17864318 | 55 | 27875 | 2 | 1 | 2 | 1 | 0.14 | ribosomal protein L8, isoform A |
| 8 | 1 | NCBInr | gi\|24583248 | 52 | 29534 | 6 | 3 | 6 | 3 | 0.44 | ribosomal protein L7 |
| 9 | 1 | NCBInr | gi\|8488 | 43 | 45727 | 9 | 3 | 9 | 3 | 0.27 | Ribosomal protein L4 (RpL4) |
| 10 | 1 | NCBInr | gi\|62862016 | 43 | 24310 | 4 | 3 | 4 | 3 | 0.55 | ribosomal protein L15, isoform A |
| 11 | 1 | NCBInr | gi\|24644770 | 40 | 69103 | 6 | 4 | 6 | 4 | 0.23 | CG10055 |
| 12 | 1 | NCBInr | gi\|17737907 | 40 | 46886 | 4 | 3 | 4 | 3 | 0.26 | ribosomal protein L3, isoform A |
| 13 | 1 | NCBInr | gi\|28574962 | 38 | 88155 | 3 | 1 | 2 | 1 | 0.04 | CG10077, isoform A |
| 14 | 1 | NCBInr | gi\|2832776 | 37 | 74931 | 2 | 1 | 2 | 1 | 0.05 | eukaryotic translation initiation factor 2B subunit epsilon |
| 15 | 1 | NCBInr | gi\|21357845 | 36 | 79184 | 3 | 1 | 3 | 1 | 0.05 | CG6904, isoform B |
| 16 | 1 | NCBInr | gi\|386764188 | 36 | 69387 | 10 | 4 | 9 | 4 | 0.23 | IGF-II mRNA-binding protein, isoform K |
| 17 | 1 | NCBInr | gi\|157594 | 32 | 51979 | 5 | 2 | 5 | 2 | 0.15 | Me31B |
| 18 | 1 | NCBInr | gi\|19920632 | 26 | 266277 | 1 | 1 | 1 | 1 | 0.01 | CG3523, isoform A |
| 19 | 1 | NCBInr | gi\|21355075 | 25 | 78519 | 3 | 1 | 3 | 1 | 0.05 | CG7878 |
| 20 | 1 | NCBInr | gi\|17647879 | 24 | 24936 | 2 | 2 | 2 | 2 | 0.33 | ribosomal protein L13, isoform A |
| 21 | 1 | NCBInr | gi\|400448 | 23 | 24020 | 1 | 1 | 1 | 1 | 0.16 | ribosomal protein L19 |
| 22 | 1 | NCBInr | gi\|17737290 | 23 | 28390 | 2 | 1 | 2 | 1 | 0.13 | ribosomal protein S6, isoform B |
| 23 | 1 | NCBInr | gi\|18858091 | 22 | 73052 | 6 | 1 | 6 | 1 | 0.05 | CG2982 |
| 24 | 1 | NCBInr | gi\|2213915 | 22 | 127265 | 2 | 1 | 2 | 1 | 0.03 | cup |
| 25 | 1 | NCBInr | gi\|45550174 | 21 | 78270 | 1 | 1 | 1 | 1 | 0.05 | CG4901 |
| 26 | 1 | NCBInr | gi\|41619560 | 20 | 23430 | 3 | 1 | 2 | 1 | 0.16 | TPA_inf: HDC14682 |
| 27 | 1 | NCBInr | gi\|21357161 | 19 | 29718 | 3 | 1 | 3 | 1 | 0.13 | ribosomal protein L6, isoform B |
| 28 | 1 | NCBInr | gi\|24651924 | 18 | 34379 | 2 | 1 | 1 | 1 | 0.11 | CG8235 |
| 29 | 1 | NCBInr | gi\|806545 | 17 | 30659 | 2 | 1 | 2 | 1 | 0.12 | ribosomal protein L7a |
| 30 | 1 | NCBInr | gi\|19921908 | 17 | 75304 | 3 | 1 | 3 | 1 | 0.05 | CG8801 |
| 31 | 1 | NCBInr | gi\|402747937 | 17 | 26361 | 3 | 1 | 2 | 1 | 0.14 | FBP2, partial |
| 32 | 1 | NCBInr | gi\|41616038 | 15 | 33214 | 7 | 1 | 1 | 1 | 0.11 | TPA_inf: HDC03427 |
| 33 | 1 | NCBInr | gi\|4375869 | 15 | 80481 | 1 | 1 | 1 | 1 | 0.05 | adrift (aft) |
| 34 | 1 | NCBInr | gi\|157981 | 13 | 124743 | 1 | 1 | 1 | 1 | 0.03 | phospholipase C |
| **S5** | | | | | | | | | | | |
| Family | Member | Database | Accession | Score | Mass | Num. of matches | Num. of significant matches | Num. of sequences | Num. of significant sequences | emPAI | Description |
| 1 | 1 | NCBInr | gi\|442631979 | 5880 | 69385 | 261 | 190 | 45 | 40 | 250.27 | trailer hitch, isoform F |
| 2 | 2 | NCBInr | gi\|442631975 | 5735 | 68675 | 252 | 183 | 41 | 36 | 172.88 | trailer hitch, isoform D |
| 3 | 1 | NCBInr | gi\|21355167 | 868 | 59706 | 46 | 36 | 30 | 23 | 5.51 | lost, isoform A |
| 4 | 1 | NCBInr | gi\|45553317 | 637 | 53644 | 36 | 25 | 20 | 13 | 4.37 | oskar, isoform C |
| 5 | 1 | NCBInr | gi\|157665 | 304 | 71015 | 26 | 17 | 20 | 13 | 1.38 | heat shock cognate 4 |
| 6 | 2 | NCBInr | gi\|157658 | 168 | 72190 | 16 | 8 | 13 | 6 | 0.49 | heat shock protein cognate 72 |
| 7 | 1 | NCBInr | gi\|386764191 | 121 | 69868 | 14 | 9 | 12 | 9 | 0.59 | IGF-II mRNA-binding protein, isoform L |
| 8 | 1 | NCBInr | gi\|2213915 | 120 | 127265 | 6 | 3 | 6 | 3 | 0.09 | cup |
| 9 | 1 | NCBInr | gi\|8488 | 99 | 45727 | 13 | 9 | 12 | 9 | 1.03 | Ribosomal protein L4 (RpL4) |
| 10 | 1 | NCBInr | gi\|21357161 | 82 | 29718 | 6 | 5 | 5 | 4 | 0.82 | ribosomal protein L6, isoform B |
| 11 | 1 | NCBInr | gi\|495594 | 80 | 69658 | 8 | 4 | 8 | 4 | 0.23 | poly(A)-binding protein |
| 12 | 1 | NCBInr | gi\|6746602 | 72 | 67297 | 4 | 4 | 4 | 4 | 0.24 | La related protein 7 (Larp7) |
| 13 | 1 | NCBInr | gi\|24583248 | 67 | 29534 | 6 | 4 | 6 | 4 | 0.62 | ribosomal protein L7 |
| 14 | 1 | NCBInr | gi\|62862016 | 65 | 24310 | 3 | 3 | 3 | 3 | 0.55 | ribosomal protein L15, isoform A |
| 15 | 1 | NCBInr | gi\|21355075 | 54 | 78519 | 7 | 5 | 7 | 5 | 0.26 | CG7878 |
| 16 | 1 | NCBInr | gi\|28574962 | 53 | 88155 | 5 | 3 | 5 | 3 | 0.13 | CG10077, isoform A |
| 17 | 1 | NCBInr | gi\|2832776 | 50 | 74931 | 3 | 3 | 3 | 3 | 0.16 | eukaryotic translation initiation factor 2B subunit epsilon |
| 18 | 1 | NCBInr | gi\|17737907 | 49 | 46886 | 3 | 2 | 3 | 2 | 0.17 | ribosomal protein L3, isoform A |
| 19 | 1 | NCBInr | gi\|22026970 | 49 | 80327 | 4 | 2 | 4 | 2 | 0.09 | Acyl-CoA synthetase long-chain, isoform B |
| 20 | 1 | NCBInr | gi\|17862576 | 44 | 78647 | 1 | 1 | 1 | 1 | 0.05 | l(3)72Dn |
| 21 | 1 | NCBInr | gi\|1511646 | 44 | 372414 | 3 | 1 | 2 | 1 | 0.01 | Apolipophorin |
| 22 | 1 | NCBInr | gi\|24651757 | 42 | 76467 | 7 | 2 | 7 | 2 | 0.1 | CG2118, isoform A |
| 23 | 1 | NCBInr | gi\|18858091 | 40 | 73052 | 7 | 4 | 7 | 4 | 0.22 | CG2982 |
| 24 | 1 | NCBInr | gi\|19921908 | 39 | 75304 | 7 | 2 | 7 | 2 | 0.1 | CG8801 |
| 25 | 1 | NCBInr | gi\|969095 | 35 | 71071 | 4 | 1 | 4 | 1 | 0.05 | no-on transient A-like protein, partial |
| 26 | 1 | NCBInr | gi\|45550712 | 32 | 71862 | 5 | 1 | 5 | 1 | 0.05 | CG9684 |
| 27 | 1 | NCBInr | gi\|158759 | 30 | 8548 | 2 | 2 | 2 | 2 | 1.18 | ubiquitin, partial |
| 28 | 1 | NCBInr | gi\|24665977 | 30 | 73370 | 2 | 1 | 2 | 1 | 0.05 | enhancer of decapping 3 |
| 29 | 1 | NCBInr | gi\|5764409 | 30 | 72508 | 2 | 1 | 2 | 1 | 0.05 | DGP-1 protein |
| 30 | 1 | NCBInr | gi\|157476 | 29 | 35294 | 1 | 1 | 1 | 1 | 0.11 | Gapdh1 |
| 31 | 1 | NCBInr | gi\|17530825 | 25 | 30713 | 6 | 3 | 5 | 3 | 0.42 | ribosomal protein L7A, isoform D |
| 32 | 1 | NCBInr | gi\|41617958 | 25 | 11490 | 1 | 1 | 1 | 1 | 0.34 | TPA_inf: HDC09524 |
| 33 | 1 | NCBInr | gi\|17737290 | 25 | 28390 | 3 | 2 | 3 | 2 | 0.28 | ribosomal protein S6, isoform B |
| 34 | 1 | NCBInr | gi\|24641509 | 24 | 36731 | 7 | 1 | 1 | 1 | 0.1 | CG32655 |
| 35 | 1 | NCBInr | gi\|24651203 | 24 | 77944 | 1 | 1 | 1 | 1 | 0.05 | CG31038, isoform A |
| 36 | 1 | NCBInr | gi\|17647879 | 24 | 24936 | 1 | 1 | 1 | 1 | 0.15 | ribosomal protein L13, isoform A |
| 37 | 1 | NCBInr | gi\|157594 | 24 | 51979 | 3 | 2 | 3 | 2 | 0.15 | Me31B |
| 38 | 1 | NCBInr | gi\|158796 | 22 | 70803 | 5 | 1 | 5 | 1 | 0.05 | Vasa |
| 39 | 1 | NCBInr | gi\|41619560 | 18 | 23430 | 6 | 3 | 2 | 1 | 0.16 | TPA_inf: HDC14682 |
| 40 | 1 | NCBInr | gi\|157981 | 18 | 124743 | 1 | 1 | 1 | 1 | 0.03 | phospholipase C |
| 41 | 1 | NCBInr | gi\|19920632 | 17 | 266277 | 1 | 1 | 1 | 1 | 0.01 | CG3523, isoform A |
| 42 | 1 | NCBInr | gi\|400448 | 16 | 24020 | 1 | 1 | 1 | 1 | 0.16 | ribosomal protein L19 |
| 43 | 1 | NCBInr | gi\|19920452 | 16 | 58592 | 1 | 1 | 1 | 1 | 0.06 | mushroom body miniature |
| 44 | 1 | NCBInr | gi\|78214297 | 16 | 103844 | 2 | 1 | 2 | 1 | 0.04 | CG17258 |
| 45 | 1 | NCBInr | gi\|24652463 | 13 | 89356 | 1 | 1 | 1 | 1 | 0.04 | withered, isoform A |
| **L6** | | | | | | | | | | | |
| Family | Member | Database | Accession | Score | Mass | Num. of matches | Num. of significant matches | Num. of sequences | Num. of significant sequences | emPAI | Description |
| 1 | 1 | NCBInr | gi\|17737967 | 1701 | 71087 | 104 | 81 | 42 | 38 | 18.18 | heat shock protein cognate 4, isoform A |
| 2 | 2 | NCBInr | gi\|157658 | 696 | 72190 | 56 | 37 | 32 | 23 | 4.23 | heat shock protein cognate 72 |
| 3 | 1 | NCBInr | gi\|21355167 | 1129 | 59706 | 77 | 52 | 39 | 31 | 10.92 | lost, isoform A |
| 4 | 1 | NCBInr | gi\|442631975 | 708 | 68675 | 39 | 27 | 24 | 20 | 2.54 | trailer hitch, isoform D |
| 5 | 1 | NCBInr | gi\|17136378 | 479 | 69882 | 43 | 26 | 31 | 21 | 2.84 | polyA-binding protein, isoform A |
| 6 | 1 | NCBInr | gi\|24640128 | 210 | 62032 | 20 | 11 | 17 | 10 | 0.9 | swallow |
| 7 | 1 | NCBInr | gi\|24584399 | 201 | 72287 | 28 | 17 | 25 | 17 | 1.34 | vasa, isoform A |
| 8 | 1 | NCBInr | gi\|386764191 | 66 | 69868 | 9 | 4 | 8 | 4 | 0.23 | IGF-II mRNA-binding protein, isoform L |
| 9 | 1 | NCBInr | gi\|158767 | 52 | 8540 | 2 | 2 | 2 | 2 | 1.18 | ubiquitin, partial |
| 10 | 1 | NCBInr | gi\|21355075 | 49 | 78519 | 6 | 4 | 6 | 4 | 0.2 | CG7878 |
| 11 | 2 | NCBInr | gi\|17985987 | 30 | 85029 | 2 | 2 | 2 | 2 | 0.09 | belle, isoform A |
| 12 | 1 | NCBInr | gi\|6118522 | 39 | 67911 | 3 | 1 | 3 | 1 | 0.05 | poly-U binding splicing factor |
| 13 | 1 | NCBInr | gi\|62862016 | 39 | 24310 | 1 | 1 | 1 | 1 | 0.16 | ribosomal protein L15, isoform A |
| 14 | 1 | NCBInr | gi\|157594 | 37 | 51979 | 4 | 2 | 4 | 2 | 0.15 | RNA helicase |
| 15 | 1 | NCBInr | gi\|24645205 | 36 | 69240 | 3 | 2 | 3 | 2 | 0.11 | oskar, isoform A |
| 16 | 1 | NCBInr | gi\|2213915 | 35 | 127265 | 1 | 1 | 1 | 1 | 0.03 | cup |
| 17 | 1 | NCBInr | gi\|19527509 | 30 | 66152 | 4 | 1 | 4 | 1 | 0.06 | peste |
| 18 | 1 | NCBInr | gi\|24651924 | 29 | 34379 | 3 | 2 | 1 | 1 | 0.11 | CG8235 |
| 19 | 1 | NCBInr | gi\|19922584 | 26 | 71582 | 1 | 1 | 1 | 1 | 0.05 | CG15118, isoform B |
| 20 | 1 | NCBInr | gi\|17647879 | 26 | 24936 | 2 | 2 | 2 | 2 | 0.33 | ribosomal protein L13, isoform A |
| 21 | 1 | NCBInr | gi\|41619560 | 24 | 23430 | 2 | 2 | 1 | 1 | 0.16 | TPA_inf: HDC14682 |
| 22 | 1 | NCBInr | gi\|78214297 | 22 | 103844 | 1 | 1 | 1 | 1 | 0.04 | CG17258 |
| 23 | 1 | NCBInr | gi\|992988 | 17 | 94896 | 1 | 1 | 1 | 1 | 0.04 | serotonin receptor 5-HT2 subtype |
| 24 | 1 | NCBInr | gi\|21356055 | 16 | 80150 | 1 | 1 | 1 | 1 | 0.05 | CG13671 |
| 25 | 1 | NCBInr | gi\|18859989 | 14 | 69458 | 2 | 1 | 2 | 1 | 0.05 | CG9281, isoform B |
| 26 | 1 | NCBInr | gi\|157667 | 13 | 74162 | 5 | 1 | 5 | 1 | 0.05 | heat shock protein cognate 71 |
| **S6** | | | | | | | | | | | |
| Family | Member | Database | Accession | Score | Mass | Num. of matches | Num. of significant matches | Num. of sequences | Num. of significant sequences | emPAI | Description |
| 1 | 1 | NCBInr | gi\|21355167 | 1915 | 59706 | 165 | 111 | 73 | 59 | 169.48 | lost, isoform A |
| 2 | 1 | NCBInr | gi\|17737967 | 1662 | 71087 | 131 | 90 | 61 | 48 | 29.33 | heat shock protein cognate 4, isoform A |
| 3 | 2 | NCBInr | gi\|157658 | 766 | 72190 | 55 | 40 | 31 | 24 | 4.5 | heat shock protein cognate 72 |
| 4 | 3 | NCBInr | gi\|17647515 | 282 | 70643 | 21 | 13 | 12 | 7 | 0.67 | heat shock protein cognate 1, isoform A |
| 5 | 1 | NCBInr | gi\|442631975 | 1294 | 68675 | 73 | 50 | 34 | 28 | 7.65 | trailer hitch, isoform D |
| 6 | 1 | NCBInr | gi\|45553317 | 646 | 53644 | 44 | 28 | 27 | 20 | 5.14 | oskar, isoform C |
| 7 | 1 | NCBInr | gi\|17136378 | 576 | 69882 | 46 | 30 | 31 | 21 | 3.26 | polyA-binding protein, isoform A |
| 8 | 1 | NCBInr | gi\|24584399 | 193 | 72287 | 27 | 17 | 25 | 16 | 1.34 | vasa, isoform A |
| 9 | 1 | NCBInr | gi\|24651389 | 125 | 75082 | 14 | 7 | 14 | 7 | 0.4 | aralar1, isoform A |
| 10 | 1 | NCBInr | gi\|21355075 | 86 | 78519 | 11 | 8 | 10 | 8 | 0.45 | CG7878 |
| 11 | 1 | NCBInr | gi\|21429908 | 33 | 44723 | 1 | 1 | 1 | 1 | 0.08 | rumpelstiltskin (rump) |
| 12 | 1 | NCBInr | gi\|6118522 | 32 | 67911 | 1 | 1 | 1 | 1 | 0.05 | poly-U binding splicing factor |
| 13 | 1 | NCBInr | gi\|19921464 | 28 | 61501 | 4 | 1 | 4 | 1 | 0.06 | CG6453, isoform A |
| 14 | 1 | NCBInr | gi\|6960212 | 24 | 63052 | 1 | 1 | 1 | 1 | 0.06 | cytoplasmic protein 89BC |
| 15 | 1 | NCBInr | gi\|19920632 | 24 | 266277 | 1 | 1 | 1 | 1 | 0.01 | CG3523, isoform A |
| 16 | 1 | NCBInr | gi\|8488 | 22 | 45727 | 1 | 1 | 1 | 1 | 0.08 | Ribosomal protein L4 (RpL4) |
| 17 | 1 | NCBInr | gi\|157981 | 21 | 124743 | 1 | 1 | 1 | 1 | 0.03 | phospholipase C |
| 18 | 1 | NCBInr | gi\|386764191 | 21 | 69868 | 7 | 3 | 7 | 3 | 0.17 | IGF-II mRNA-binding protein, isoform L |
| 19 | 1 | NCBInr | gi\|402747937 | 20 | 26361 | 4 | 2 | 3 | 2 | 0.31 | FBP2, partial |
| 20 | 1 | NCBInr | gi\|24651924 | 20 | 34379 | 2 | 1 | 1 | 1 | 0.11 | CG8235 |
| 21 | 1 | NCBInr | gi\|16183808 | 13 | 70961 | 1 | 1 | 1 | 1 | 0.05 | Zwilch |
| **L7** | | | | | | | | | | | |
| Family | Member | Database | Accession | Score | Mass | Num. of matches | Num. of significant matches | Num. of sequences | Num. of significant sequences | emPAI | Description |
| 1 | 1 | NCBInr | gi\|17136378 | 822 | 69882 | 42 | 33 | 27 | 23 | 3.97 | polyA-binding protein, isoform A |
| 2 | 1 | NCBInr | gi\|17530805 | 151 | 41795 | 8 | 8 | 7 | 7 | 0.99 | actin |
| 3 | 2 | NCBInr | gi\|156763 | 72 | 41778 | 6 | 5 | 6 | 5 | 0.54 | actin |
| 4 | 1 | NCBInr | gi\|6960212 | 103 | 63052 | 8 | 5 | 8 | 5 | 0.33 | cytoplasmic protein 89BC |
| 5 | 1 | NCBInr | gi\|45550607 | 84 | 69298 | 3 | 3 | 3 | 3 | 0.17 | trailer hitch, isoform A |
| 6 | 1 | NCBInr | gi\|6691133 | 81 | 47305 | 2 | 2 | 1 | 1 | 0.08 | SP295; Thrombospondin (Tsp) |
| 7 | 1 | NCBInr | gi\|7915 | 73 | 50250 | 3 | 2 | 3 | 2 | 0.15 | EF-1-alpha |
| 8 | 1 | NCBInr | gi\|17136376 | 72 | 91095 | 4 | 2 | 3 | 2 | 0.08 | armadillo, isoform A |
| 9 | 1 | NCBInr | gi\|157476 | 53 | 35294 | 1 | 1 | 1 | 1 | 0.11 | Gadph-1 |
| 10 | 1 | NCBInr | gi\|6467825 | 52 | 596754 | 3 | 1 | 3 | 1 | 0.01 | Spen RNP motif protein long isoform |
| 11 | 1 | NCBInr | gi\|21429908 | 51 | 44723 | 2 | 2 | 2 | 2 | 0.17 | rumpelstiltskin (rump) |
| 12 | 1 | NCBInr | gi\|17157991 | 41 | 21724 | 2 | 2 | 2 | 2 | 0.38 | thioredoxin peroxidase 1, isoform A |
| 13 | 1 | NCBInr | gi\|24647058 | 41 | 60556 | 1 | 1 | 1 | 1 | 0.06 | CG14864, isoform A |
| 14 | 1 | NCBInr | gi\|24640128 | 35 | 62032 | 2 | 2 | 2 | 2 | 0.12 | swallow |
| 15 | 1 | NCBInr | gi\|18859877 | 34 | 26764 | 1 | 1 | 1 | 1 | 0.14 | CG5703 |
| 16 | 1 | NCBInr | gi\|41619560 | 27 | 23430 | 32 | 4 | 1 | 1 | 0.16 | TPA_inf: HDC14682 |
| 17 | 1 | NCBInr | gi\|24649524 | 27 | 50457 | 1 | 1 | 1 | 1 | 0.07 | CG5991, isoform A |
| 18 | 1 | NCBInr | gi\|21355167 | 26 | 59706 | 5 | 2 | 5 | 2 | 0.13 | lost, isoform A |
| 19 | 1 | NCBInr | gi\|19920632 | 26 | 266277 | 3 | 1 | 2 | 1 | 0.01 | CG3523, isoform A |
| 20 | 1 | NCBInr | gi\|158759 | 24 | 8548 | 1 | 1 | 1 | 1 | 0.48 | ubiquitin, partial |
| 21 | 1 | NCBInr | gi\|78706658 | 24 | 18877 | 2 | 1 | 1 | 1 | 0.2 | CG33689 |
| 22 | 1 | NCBInr | gi\|78214297 | 19 | 103844 | 2 | 1 | 2 | 1 | 0.04 | CG17258 |
| 23 | 1 | NCBInr | gi\|992988 | 16 | 94896 | 1 | 1 | 1 | 1 | 0.04 | serotonin receptor 5-HT2 subtype |
| 24 | 1 | NCBInr | gi\|429892566 | 13 | 164336 | 2 | 1 | 1 | 1 | 0.02 | spindle E |
| **S7** | | | | | | | | | | | |
| Family | Member | Database | Accession | Score | Mass | Num. of matches | Num. of significant matches | Num. of sequences | Num. of significant sequences | emPAI | Description |
| 1 | 1 | NCBInr | gi\|17136378 | 1146 | 69882 | 91 | 51 | 40 | 30 | 8.25 | polyA-binding protein, isoform A |
| 2 | 1 | NCBInr | gi\|45553317 | 1084 | 53644 | 65 | 47 | 25 | 24 | 12.77 | oskar, isoform C |
| 3 | 1 | NCBInr | gi\|21355167 | 318 | 59706 | 23 | 18 | 21 | 17 | 1.97 | lost, isoform A |
| 4 | 1 | NCBInr | gi\|442631973 | 279 | 69711 | 26 | 13 | 22 | 10 | 0.86 | trailer hitch, isoform C |
| 5 | 1 | NCBInr | gi\|18859989 | 272 | 69458 | 27 | 16 | 20 | 13 | 1.3 | CG9281, isoform B |
| 6 | 1 | NCBInr | gi\|24645384 | 265 | 66691 | 18 | 14 | 13 | 11 | 1.14 | rumpelstiltskin, isoform A |
| 7 | 1 | NCBInr | gi\|24651387 | 147 | 76706 | 14 | 9 | 14 | 9 | 0.53 | aralar1, isoform C |
| 8 | 1 | NCBInr | gi\|17530887 | 120 | 62091 | 20 | 12 | 16 | 11 | 1.01 | IGF-II mRNA-binding protein, isoform A |
| 9 | 1 | NCBInr | gi\|16198217 | 115 | 60575 | 14 | 10 | 13 | 10 | 0.81 | Apoptosis inducing factor (AIF) |
| 10 | 1 | NCBInr | gi\|19527547 | 113 | 68233 | 11 | 6 | 11 | 6 | 0.37 | Vacuolar H+ ATPase 68 kDa subunit 2 (Vha68-2) |
| 11 | 1 | NCBInr | gi\|7141239 | 112 | 63772 | 10 | 6 | 9 | 5 | 0.4 | eukaryotic translation initiation factor 3 p66 subunit |
| 12 | 1 | NCBInr | gi\|2148976 | 107 | 64182 | 13 | 8 | 11 | 7 | 0.57 | bruno |
| 13 | 1 | NCBInr | gi\|24653318 | 105 | 74160 | 15 | 8 | 14 | 8 | 0.48 | CG4679 |
| 14 | 1 | NCBInr | gi\|18858123 | 88 | 64867 | 10 | 6 | 10 | 6 | 0.4 | spoonbill, isoform A |
| 15 | 1 | NCBInr | gi\|17530957 | 81 | 65067 | 5 | 2 | 4 | 2 | 0.12 | mitochondrial ribosomal protein S30 |
| 16 | 1 | NCBInr | gi\|494737 | 60 | 16669 | 3 | 1 | 2 | 1 | 0.23 | Calmodulin (Cam) |
| 17 | 1 | NCBInr | gi\|8488 | 56 | 45727 | 7 | 3 | 7 | 3 | 0.27 | Ribosomal protein L4 (RpL4) |
| 18 | 1 | NCBInr | gi\|62862016 | 48 | 24310 | 4 | 3 | 4 | 3 | 0.55 | ribosomal protein L15, isoform A |
| 19 | 1 | NCBInr | gi\|17975542 | 43 | 11374 | 1 | 1 | 1 | 1 | 0.35 | histone H4 replacement, isoform C |
| 20 | 1 | NCBInr | gi\|18543229 | 42 | 70202 | 7 | 2 | 7 | 2 | 0.11 | nucleostemin 3 |
| 21 | 1 | NCBInr | gi\|157665 | 40 | 71015 | 7 | 4 | 7 | 4 | 0.23 | heat shock cognate 4 |
| 22 | 1 | NCBInr | gi\|19922420 | 38 | 63190 | 3 | 1 | 3 | 1 | 0.06 | CG8963, isoform A |
| 23 | 1 | NCBInr | gi\|28574962 | 34 | 88155 | 5 | 2 | 5 | 2 | 0.09 | CG10077, isoform A |
| 24 | 2 | NCBInr | gi\|17985987 | 23 | 85029 | 5 | 2 | 5 | 2 | 0.09 | belle, isoform A |
| 25 | 1 | NCBInr | gi\|161076325 | 33 | 183829 | 3 | 1 | 3 | 1 | 0.02 | eukaryotic translation initiation factor 4G, isoform A |
| 26 | 1 | NCBInr | gi\|1813955 | 28 | 54909 | 2 | 1 | 2 | 1 | 0.07 | succinate dehydrogenase flavoprotein subunit |
| 27 | 1 | NCBInr | gi\|24640849 | 26 | 64620 | 6 | 2 | 6 | 2 | 0.12 | Lysyl-tRNA synthetase, isoform A |
| 28 | 1 | NCBInr | gi\|19920632 | 25 | 266277 | 1 | 1 | 1 | 1 | 0.01 | CG3523, isoform A |
| 29 | 1 | NCBInr | gi\|24642055 | 21 | 71370 | 2 | 1 | 2 | 1 | 0.05 | CG12398 |
| 30 | 1 | NCBInr | gi\|21355559 | 20 | 69041 | 2 | 1 | 2 | 1 | 0.05 | CG9630 |
| 31 | 1 | NCBInr | gi\|19922816 | 20 | 67570 | 2 | 2 | 2 | 2 | 0.11 | eIF2B-delta, isoform A |
| 32 | 1 | NCBInr | gi\|41619560 | 20 | 23430 | 3 | 1 | 1 | 1 | 0.16 | TPA_inf: HDC14682 |
| 33 | 1 | NCBInr | gi\|158767 | 19 | 8540 | 1 | 1 | 1 | 1 | 0.48 | ubiquitin, partial |
| 34 | 1 | NCBInr | gi\|92109894 | 18 | 43066 | 1 | 1 | 1 | 1 | 0.09 | Down syndrome cell adhesion molecule 4 (Dscam4) |
| 35 | 1 | NCBInr | gi\|24640128 | 17 | 62032 | 5 | 2 | 5 | 2 | 0.12 | swallow |
| 36 | 1 | NCBInr | gi\|24580706 | 15 | 34522 | 1 | 1 | 1 | 1 | 0.11 | peroxin 12, isoform A |
| 37 | 1 | NCBInr | gi\|21357161 | 15 | 29718 | 2 | 2 | 2 | 2 | 0.27 | ribosomal protein L6, isoform B |
| 38 | 1 | NCBInr | gi\|17737907 | 15 | 46886 | 2 | 1 | 2 | 1 | 0.08 | ribosomal protein L3, isoform A |
| 39 | 1 | NCBInr | gi\|157981 | 15 | 124743 | 1 | 1 | 1 | 1 | 0.03 | phospholipase C |
| 40 | 1 | NCBInr | gi\|24649986 | 14 | 61393 | 1 | 1 | 1 | 1 | 0.06 | CG11859 |
| 41 | 1 | NCBInr | gi\|24667063 | 14 | 67340 | 1 | 1 | 1 | 1 | 0.06 | precursor RNA processing 3, isoform A |
| **L8** | | | | | | | | | | | |
| Family | Member | Database | Accession | Score | Mass | Num. of matches | Num. of significant matches | Num. of sequences | Num. of significant sequences | emPAI | Description |
| 1 | 1 | NCBInr | gi\|24583279 | 3176 | 51912 | 185 | 138 | 50 | 41 | 238.65 | maternal expression at 31B, isoform A |
| 2 | 1 | NCBInr | gi\|24655737 | 618 | 50115 | 44 | 33 | 23 | 20 | 5.04 | beta-Tubulin at 56D, isoform B |
| 3 | 2 | NCBInr | gi\|158743 | 389 | 49812 | 32 | 23 | 16 | 12 | 1.96 | beta-2 tubulin |
| 4 | 1 | NCBInr | gi\|24663131 | 549 | 38158 | 32 | 27 | 18 | 17 | 6.17 | ypsilon schachtel, isoform A |
| 5 | 1 | NCBInr | gi\|17136564 | 357 | 49876 | 24 | 16 | 14 | 11 | 1.96 | alpha-Tubulin at 84B |
| 6 | 1 | NCBInr | gi\|156763 | 128 | 41778 | 7 | 6 | 6 | 6 | 0.68 | actin |
| 7 | 2 | NCBInr | gi\|156773 | 105 | 41748 | 5 | 5 | 5 | 5 | 0.54 | actin |
| 8 | 1 | NCBInr | gi\|17738151 | 138 | 47777 | 14 | 11 | 13 | 10 | 1.29 | Tat-binding protein-1 |
| 9 | 1 | NCBInr | gi\|1072120 | 128 | 42755 | 13 | 7 | 10 | 6 | 0.66 | nucleosome assembly protein NAP-1 |
| 10 | 1 | NCBInr | gi\|8488 | 106 | 45727 | 12 | 6 | 11 | 6 | 0.6 | Ribosomal protein L4 (RpL4) |
| 11 | 1 | NCBInr | gi\|24645205 | 90 | 69240 | 10 | 6 | 9 | 6 | 0.37 | oskar, isoform A |
| 12 | 1 | NCBInr | gi\|495594 | 87 | 69658 | 9 | 4 | 9 | 4 | 0.23 | poly(A)-binding protein |
| 13 | 1 | NCBInr | gi\|1945346 | 79 | 51234 | 9 | 5 | 9 | 5 | 0.42 | gamma-tubulin |
| 14 | 1 | NCBInr | gi\|8484 | 73 | 28188 | 6 | 3 | 6 | 3 | 0.46 | ribosomal protein |
| 15 | 1 | NCBInr | gi\|17136324 | 69 | 27454 | 5 | 3 | 5 | 3 | 0.48 | ribosomal protein S3 |
| 16 | 1 | NCBInr | gi\|7915 | 68 | 50250 | 6 | 5 | 6 | 5 | 0.43 | EF-1-alpha |
| 17 | 1 | NCBInr | gi\|17647879 | 65 | 24936 | 5 | 3 | 5 | 3 | 0.53 | ribosomal protein L13, isoform A |
| 18 | 1 | NCBInr | gi\|17864318 | 65 | 27875 | 4 | 2 | 4 | 2 | 0.29 | ribosomal protein L8, isoform A |
| 19 | 1 | NCBInr | gi\|21357161 | 65 | 29718 | 3 | 3 | 2 | 2 | 0.43 | ribosomal protein L6, isoform B |
| 20 | 1 | NCBInr | gi\|62862016 | 61 | 24310 | 6 | 4 | 6 | 4 | 0.79 | ribosomal protein L15, isoform A |
| 21 | 1 | NCBInr | gi\|17737290 | 61 | 28390 | 3 | 3 | 3 | 3 | 0.46 | ribosomal protein S6, isoform B |
| 22 | 1 | NCBInr | gi\|11042 | 54 | 41004 | 3 | 2 | 3 | 2 | 0.19 | hrp48.1 |
| 23 | 1 | NCBInr | gi\|7637 | 51 | 42500 | 5 | 3 | 5 | 3 | 0.29 | 52-kD bracketing protein |
| 24 | 1 | NCBInr | gi\|7243680 | 48 | 50184 | 3 | 2 | 3 | 2 | 0.15 | pontin |
| 25 | 1 | NCBInr | gi\|158767 | 45 | 8540 | 2 | 2 | 2 | 2 | 1.18 | ubiquitin, partial |
| 26 | 1 | NCBInr | gi\|17864182 | 41 | 20604 | 2 | 1 | 2 | 1 | 0.19 | ADP ribosylation factor 102F, isoform A |
| 27 | 1 | NCBInr | gi\|287945 | 39 | 53487 | 6 | 4 | 6 | 4 | 0.31 | ATP synthase beta subunit |
| 28 | 1 | NCBInr | gi\|17136376 | 39 | 91095 | 2 | 1 | 2 | 1 | 0.04 | armadillo, isoform A |
| 29 | 1 | NCBInr | gi\|464020 | 37 | 44894 | 3 | 1 | 3 | 1 | 0.08 | La/SS-B |
| 30 | 1 | NCBInr | gi\|24647885 | 36 | 29780 | 3 | 2 | 3 | 2 | 0.27 | 14-3-3epsilon, isoform A |
| 31 | 1 | NCBInr | gi\|17136764 | 31 | 46626 | 3 | 2 | 3 | 2 | 0.17 | U2 small nuclear riboprotein auxiliary factor 50, isoform A |
| 32 | 1 | NCBInr | gi\|20130121 | 30 | 52496 | 3 | 2 | 3 | 2 | 0.15 | CG5190 |
| 33 | 1 | NCBInr | gi\|119508284 | 29 | 48210 | 3 | 2 | 3 | 2 | 0.16 | CG4951 |
| 34 | 1 | NCBInr | gi\|24663668 | 29 | 29116 | 3 | 2 | 3 | 2 | 0.28 | ribosomal protein S4, isoform A |
| 35 | 1 | NCBInr | gi\|28572126 | 27 | 54803 | 3 | 1 | 3 | 1 | 0.07 | Nop56 |
| 36 | 1 | NCBInr | gi\|17737907 | 27 | 46886 | 3 | 1 | 3 | 1 | 0.08 | ribosomal protein L3, isoform A |
| 37 | 1 | NCBInr | gi\|17157991 | 24 | 21724 | 1 | 1 | 1 | 1 | 0.18 | thioredoxin peroxidase 1, isoform A |
| 38 | 1 | NCBInr | gi\|24651203 | 24 | 77944 | 1 | 1 | 1 | 1 | 0.05 | CG31038, isoform A |
| 39 | 1 | NCBInr | gi\|41619560 | 21 | 23430 | 2 | 1 | 1 | 1 | 0.16 | TPA_inf: HDC14682 |
| 40 | 1 | NCBInr | gi\|494737 | 20 | 16669 | 1 | 1 | 1 | 1 | 0.23 | Calmodulin (Cam) |
| 41 | 1 | NCBInr | gi\|17136734 | 20 | 28881 | 1 | 1 | 1 | 1 | 0.13 | ribosomal protein S2, isoform A |
| 42 | 1 | NCBInr | gi\|21357053 | 20 | 23632 | 2 | 1 | 2 | 1 | 0.16 | ribosomal protein L13A, isoform B |
| 43 | 1 | NCBInr | gi\|429892566 | 20 | 164336 | 1 | 1 | 1 | 1 | 0.02 | spindle E |
| 44 | 1 | NCBInr | gi\|6014919 | 19 | 51283 | 1 | 1 | 1 | 1 | 0.07 | Dead box protein 80 (Dbp80) |
| 45 | 1 | NCBInr | gi\|78706658 | 19 | 18877 | 2 | 1 | 1 | 1 | 0.2 | CG33689 |
| 46 | 1 | NCBInr | gi\|24639780 | 18 | 59634 | 2 | 1 | 2 | 1 | 0.06 | CG6927 |
| 47 | 1 | NCBInr | gi\|15291817 | 18 | 112804 | 1 | 1 | 1 | 1 | 0.03 | CG2691 |
| 48 | 1 | NCBInr | gi\|17647881 | 18 | 19162 | 2 | 1 | 2 | 1 | 0.2 | ribosomal protein L14 |
| 49 | 1 | NCBInr | gi\|18079273 | 18 | 32888 | 4 | 1 | 4 | 1 | 0.11 | stress-sensitive B, isoform A |
| 50 | 1 | NCBInr | gi\|19527857 | 15 | 50220 | 1 | 1 | 1 | 1 | 0.07 | Ubiquitin-like activating enzyme 4 (Uba4) |
| 51 | 1 | NCBInr | gi\|402747937 | 15 | 26361 | 3 | 1 | 2 | 1 | 0.14 | FBP2, partial |
| 52 | 1 | NCBInr | gi\|400448 | 15 | 24020 | 2 | 1 | 2 | 1 | 0.16 | ribosomal protein L19 |
| 53 | 1 | NCBInr | gi\|19920632 | 14 | 266277 | 1 | 1 | 1 | 1 | 0.01 | CG3523, isoform A |
| 54 | 1 | NCBInr | gi\|1168016 | 13 | 42576 | 1 | 1 | 1 | 1 | 0.09 | 26S protease regulatory complex non-ATPase subunit |
| **S8** | | | | | | | | | | | |
| Family | Member | Database | Accession | Score | Mass | Num. of matches | Num. of significant matches | Num. of sequences | Num. of significant sequences | emPAI | Description |
| 1 | 1 | NCBInr | gi\|24583279 | 7303 | 51912 | 402 | 293 | 64 | 54 | 3839.73 | maternal expression at 31B, isoform A |
| 2 | 1 | NCBInr | gi\|24655737 | 1012 | 50115 | 59 | 44 | 27 | 22 | 8.99 | beta-Tubulin at 56D, isoform B |
| 3 | 1 | NCBInr | gi\|24663131 | 716 | 38158 | 44 | 34 | 21 | 18 | 7.66 | ypsilon schachtel, isoform A |
| 4 | 1 | NCBInr | gi\|17136564 | 535 | 49876 | 34 | 21 | 17 | 13 | 2.95 | alpha-Tubulin at 84B |
| 5 | 1 | NCBInr | gi\|45553317 | 410 | 53644 | 24 | 16 | 16 | 9 | 1.93 | oskar, isoform C |
| 6 | 1 | NCBInr | gi\|287945 | 341 | 53487 | 24 | 19 | 17 | 15 | 2.6 | ATP synthase beta subunit |
| 7 | 1 | NCBInr | gi\|1945346 | 258 | 51234 | 14 | 10 | 13 | 10 | 1.02 | gamma-tubulin |
| 8 | 1 | NCBInr | gi\|17136378 | 223 | 69882 | 23 | 10 | 20 | 10 | 0.68 | polyA-binding protein, isoform A |
| 9 | 1 | NCBInr | gi\|24649014 | 179 | 49824 | 14 | 10 | 14 | 10 | 1.06 | AP-50, isoform A |
| 10 | 1 | NCBInr | gi\|17738151 | 171 | 47777 | 17 | 9 | 14 | 8 | 0.97 | Tat-binding protein-1 |
| 11 | 1 | NCBInr | gi\|28572126 | 168 | 54803 | 12 | 9 | 11 | 8 | 0.81 | Nop56 |
| 12 | 1 | NCBInr | gi\|21358125 | 168 | 50211 | 9 | 8 | 9 | 8 | 0.78 | pontin |
| 13 | 1 | NCBInr | gi\|1072120 | 150 | 42755 | 21 | 14 | 14 | 11 | 1.74 | nucleosome assembly protein NAP-1 |
| 14 | 1 | NCBInr | gi\|45550607 | 141 | 69298 | 12 | 8 | 12 | 8 | 0.52 | trailer hitch, isoform A |
| 15 | 1 | NCBInr | gi\|8488 | 121 | 45727 | 13 | 8 | 12 | 8 | 0.88 | Ribosomal protein L4 (RpL4) |
| 16 | 1 | NCBInr | gi\|11042 | 117 | 41004 | 8 | 3 | 7 | 3 | 0.3 | hrp48.1 |
| 17 | 1 | NCBInr | gi\|24583248 | 92 | 29534 | 12 | 5 | 12 | 5 | 0.83 | ribosomal protein L7 |
| 18 | 1 | NCBInr | gi\|464020 | 84 | 44894 | 11 | 5 | 9 | 4 | 0.49 | La/SS-B |
| 19 | 1 | NCBInr | gi\|17864358 | 76 | 53958 | 8 | 5 | 8 | 5 | 0.4 | elongation factor Tu mitochondrial, isoform A |
| 20 | 1 | NCBInr | gi\|7637 | 71 | 42500 | 8 | 3 | 6 | 3 | 0.29 | 52-kD bracketing protein |
| 21 | 1 | NCBInr | gi\|21357161 | 69 | 29718 | 3 | 3 | 2 | 2 | 0.43 | ribosomal protein L6, isoform B |
| 22 | 1 | NCBInr | gi\|2564707 | 67 | 51602 | 4 | 3 | 4 | 3 | 0.23 | maelstrom |
| 23 | 1 | NCBInr | gi\|17737907 | 65 | 46886 | 12 | 6 | 10 | 5 | 0.59 | ribosomal protein L3, isoform A |
| 24 | 1 | NCBInr | gi\|241021 | 65 | 57939 | 5 | 2 | 5 | 2 | 0.13 | exuperantia |
| 25 | 1 | NCBInr | gi\|17737290 | 50 | 28390 | 4 | 3 | 4 | 3 | 0.46 | ribosomal protein S6, isoform B |
| 26 | 1 | NCBInr | gi\|15292571 | 48 | 50064 | 2 | 2 | 2 | 2 | 0.15 | CG5913 |
| 27 | 1 | NCBInr | gi\|21355167 | 48 | 59706 | 2 | 2 | 2 | 2 | 0.13 | lost, isoform A |
| 28 | 1 | NCBInr | gi\|17137572 | 47 | 50275 | 9 | 3 | 8 | 3 | 0.24 | elongation factor 1alpha48D, isoform A |
| 29 | 1 | NCBInr | gi\|62862016 | 42 | 24310 | 5 | 4 | 5 | 4 | 0.79 | ribosomal protein L15, isoform A |
| 30 | 1 | NCBInr | gi\|62862344 | 37 | 52171 | 10 | 2 | 10 | 2 | 0.15 | eukaryotic initiation factor 4B, isoform B |
| 31 | 1 | NCBInr | gi\|24641079 | 35 | 58148 | 4 | 3 | 4 | 3 | 0.2 | transport and golgi organization 5, isoform A |
| 32 | 1 | NCBInr | gi\|6014919 | 34 | 51283 | 3 | 3 | 3 | 3 | 0.23 | Dead box protein 80 (Dbp80) |
| 33 | 1 | NCBInr | gi\|20130121 | 33 | 52496 | 5 | 3 | 5 | 3 | 0.23 | CG5190 |
| 34 | 1 | NCBInr | gi\|290260 | 32 | 50760 | 5 | 2 | 5 | 2 | 0.15 | phosphoprotein phosphatase 2A 55 kDa regulatory subunit |
| 35 | 1 | NCBInr | gi\|17530825 | 30 | 30713 | 8 | 4 | 8 | 4 | 0.59 | ribosomal protein L7A, isoform D |
| 36 | 1 | NCBInr | gi\|24647046 | 28 | 51452 | 7 | 2 | 7 | 2 | 0.15 | eukaryotic initiation factor 2gamma, isoform B |
| 37 | 1 | NCBInr | gi\|24661707 | 28 | 22610 | 5 | 2 | 5 | 2 | 0.37 | ribosomal protein S9, isoform A |
| 38 | 1 | NCBInr | gi\|24651203 | 27 | 77944 | 2 | 1 | 1 | 1 | 0.05 | CG31038, isoform A |
| 39 | 1 | NCBInr | gi\|1168016 | 27 | 42576 | 5 | 2 | 5 | 2 | 0.18 | 26S protease regulatory complex non-ATPase subunit |
| 40 | 1 | NCBInr | gi\|2500374 | 26 | 16040 | 1 | 1 | 1 | 1 | 0.24 | Ribosomal protein L32 (RpL32) |
| 41 | 1 | NCBInr | gi\|19527557 | 25 | 26428 | 1 | 1 | 1 | 1 | 0.14 | NADH dehydrogenase (ubiquinone) 51 kDa subunit (ND-51) |
| 42 | 1 | NCBInr | gi\|11559596 | 25 | 50653 | 3 | 2 | 3 | 2 | 0.15 | eukaryotic initiation factor eIF2B gamma subunit |
| 43 | 1 | NCBInr | gi\|17647879 | 24 | 24936 | 1 | 1 | 1 | 1 | 0.15 | ribosomal protein L13, isoform A |
| 44 | 1 | NCBInr | gi\|19921026 | 22 | 53681 | 5 | 2 | 5 | 2 | 0.14 | CG13126 |
| 45 | 1 | NCBInr | gi\|17530887 | 21 | 62091 | 3 | 1 | 3 | 1 | 0.06 | IGF-II mRNA-binding protein, isoform A |
| 46 | 1 | NCBInr | gi\|158767 | 21 | 8540 | 2 | 1 | 2 | 1 | 0.48 | ubiquitin, partial |
| 47 | 1 | NCBInr | gi\|17933648 | 19 | 48605 | 4 | 1 | 4 | 1 | 0.08 | chromatin assembly factor 1 subunit, isoform A |
| 48 | 1 | NCBInr | gi\|992988 | 19 | 94896 | 1 | 1 | 1 | 1 | 0.04 | serotonin receptor 5-HT2 subtype |
| 49 | 1 | NCBInr | gi\|41619560 | 18 | 23430 | 2 | 1 | 1 | 1 | 0.16 | TPA_inf: HDC14682 |
| 50 | 1 | NCBInr | gi\|24653276 | 17 | 52500 | 1 | 1 | 1 | 1 | 0.07 | serine palmitoyltransferase subunit I, isoform A |
| 51 | 1 | NCBInr | gi\|24649935 | 16 | 48676 | 3 | 1 | 3 | 1 | 0.08 | vig2, isoform B |
| 52 | 1 | NCBInr | gi\|24661864 | 15 | 42489 | 2 | 1 | 2 | 1 | 0.09 | CG18178, isoform A |
| 53 | 1 | NCBInr | gi\|17946318 | 15 | 18031 | 2 | 1 | 2 | 1 | 0.21 | Ribosomal protein L34a (RpL34a) |
| 54 | 1 | NCBInr | gi\|21357147 | 14 | 46892 | 2 | 1 | 2 | 1 | 0.08 | CstF-50 |
