# Supplementary material for: Receptor-mediated yolk uptake is required for oskar mRNA localization and cortical anchorage of germ plasm components in the Drosophila oocyte
Source: PLoS Biol. 2021 Apr 23;19(4):e3001183. doi: 10.1371/journal.pbio.3001183 (PMC8064586; doi:10.1371/journal.pbio.3001183)
Supplement: S2 Table — (DOCX) [file pbio.3001183.s008.docx]

**S2 Table. Oligo DNA pairs for sgRNA expression from pDCC6 plasmid**

| **Oligo name** | **Forward Sequence (5'-3')** | **Reverse Sequence (5'-3')** |
| --- | --- | --- |
| *yl*-21F & 40R | cttcgTTTGCTCGCTGGGGTGCACC | aaacGGTGCACCCCAGCGAGCAAAc |
| *yl*-48F & 67R | cttcgTCATCTTCGGGCTCTCTACC | aaacGGTAGAGAGCCCGAAGATGAc |
| *yl*-98F & 117R | cttcgTGGGTGAGCGCGTGTCTGCG | aaacCGCAGACACGCGCTCACCCAc |
| *yp1*-72F & 91R | cttcgGGTTGACGGAGTTGTCCATA | aaacTATGGACAACTCCGTCAACCc |
| *yp2*-129F & 148R | cttcgGGTCAATCCACGTGAAGTCG | aaacCGACTTCACGTGGATTGACCc |
| *yp2*-204F & 223R | cttcgGGAGGAGGGCGCTACGCTGT | aaacACAGCGTAGCGCCCTCCTCCc |
| *yp3*-53F & 72R | cttcgGGAGGCATCCTTGGAGGCAT | aaacATGCCTCCAAGGATGCCTCCc |
| *yp3*-74F & 93R | cttcgGGTCGGCTTCAGTCGGTCAT | aaacATGACCGACTGAAGCCGACCc |
| *apolpp*-8F & 27R | cttcgAGCGATATTGTATTTCATCC | aaacGGATGAAATACAATATCGCTc |
| *apolpp*-42F & 61R | cttcgTTGTTAAAAGCACAGAAGCT | aaacAGCTTCTGTGCTTTTAACAAc |
