# Supplementary material for: Receptor-mediated yolk uptake is required for oskar mRNA localization and cortical anchorage of germ plasm components in the Drosophila oocyte
Source: PLoS Biol. 2021 Apr 23;19(4):e3001183. doi: 10.1371/journal.pbio.3001183 (PMC8064586; doi:10.1371/journal.pbio.3001183)

An uncropped gel image presented in Fig 1A and S1C Fig

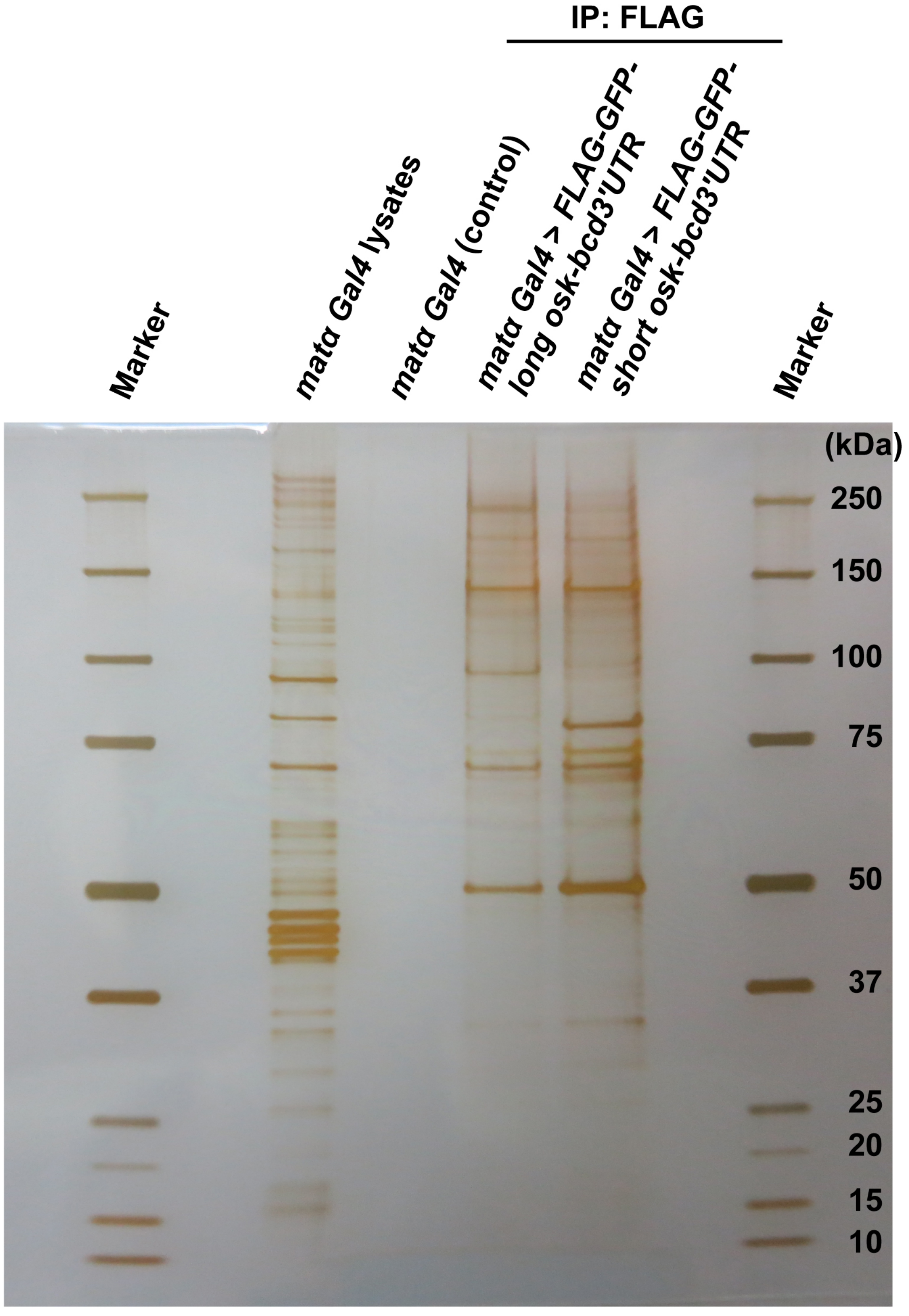

Uncropped blot images presented in Fig 1B

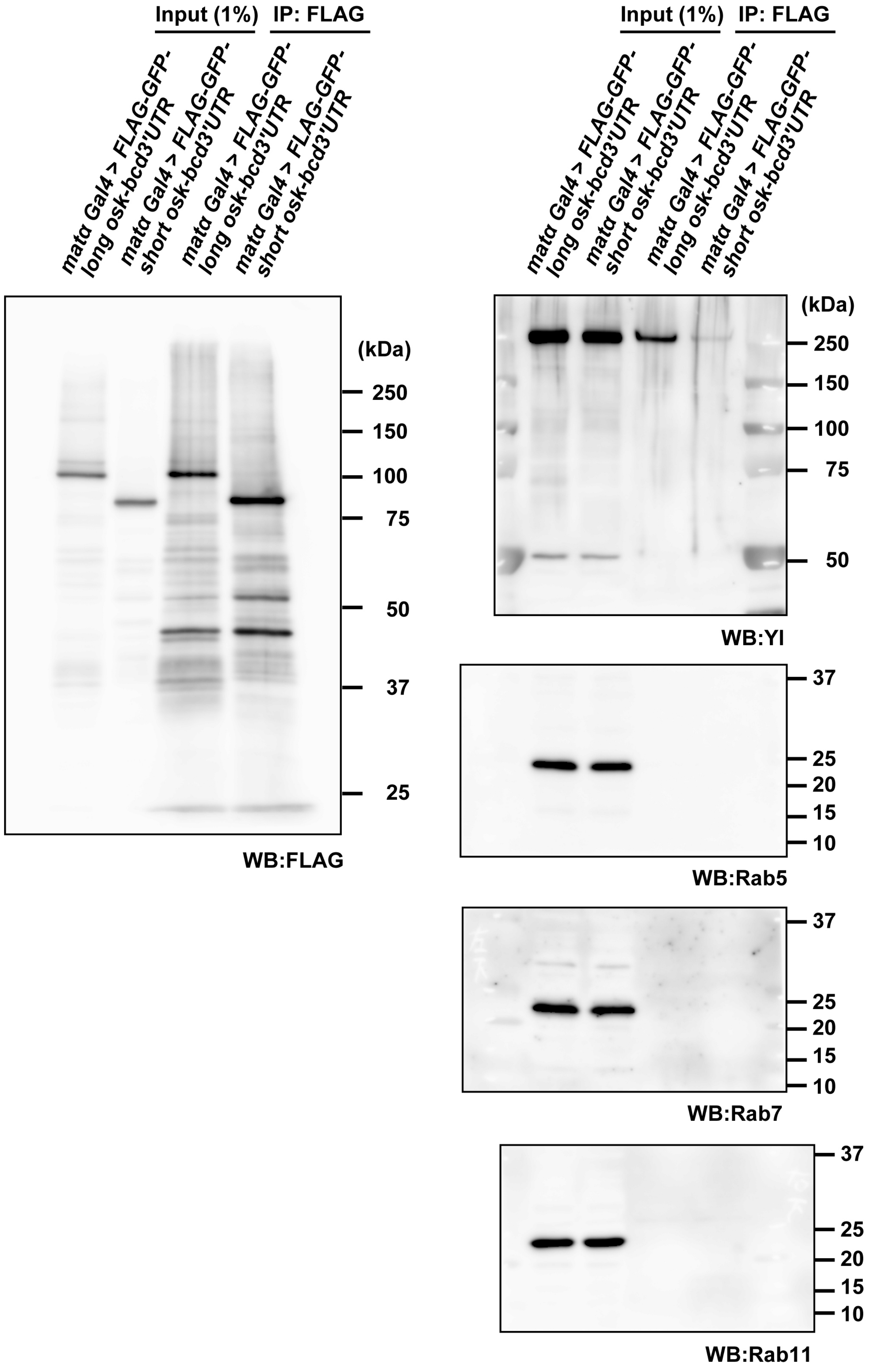

An uncropped gel image presented in Fig 1F

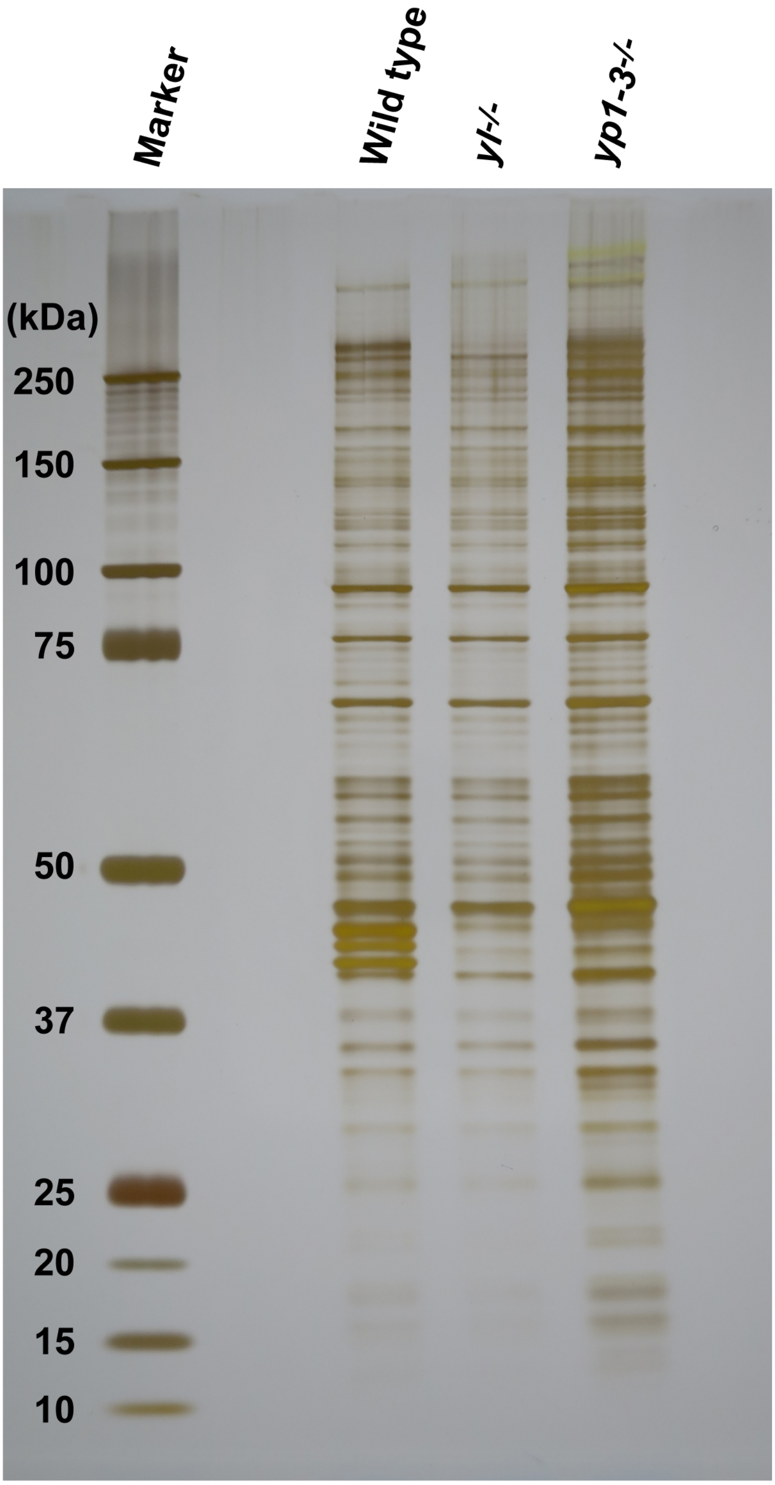

Uncropped blot images presented in S2C Fig

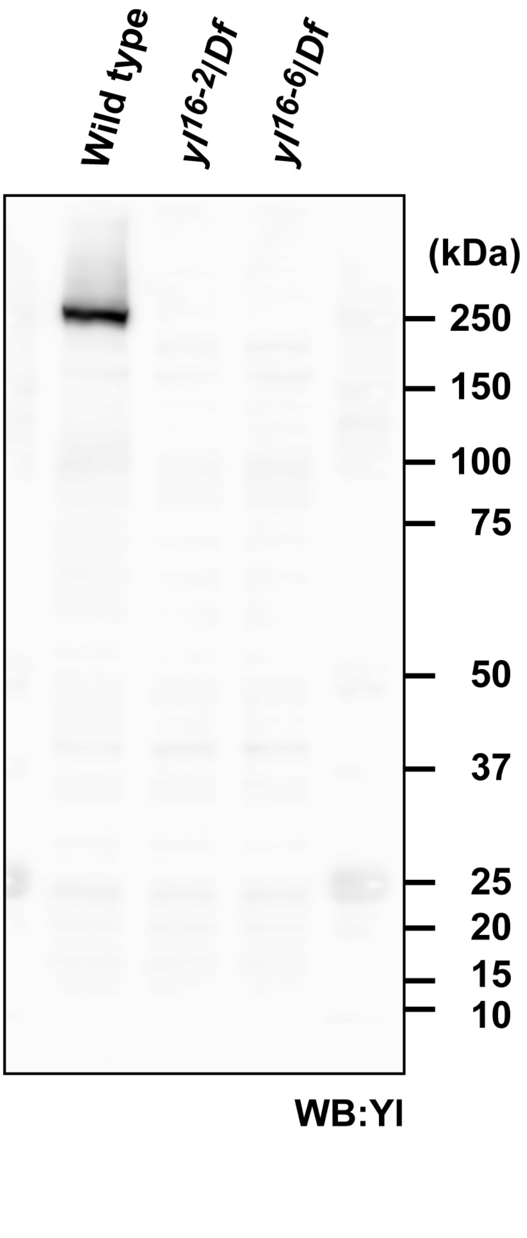

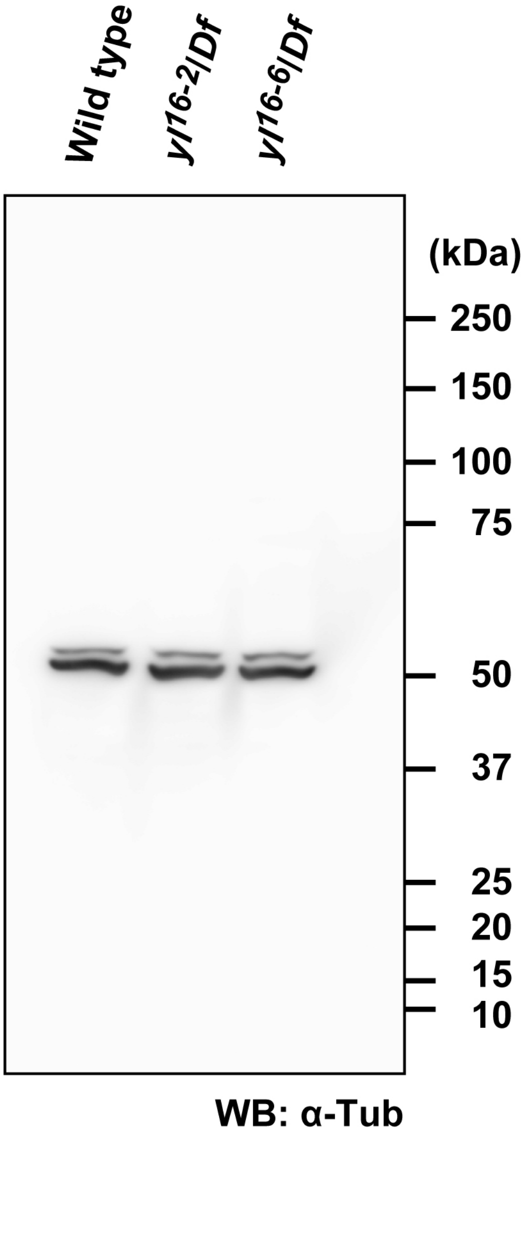

Supplement: S1 Raw Images — (PDF) [file pbio.3001183.s010.pdf]
