# Supplementary material for: Receptor-mediated yolk uptake is required for oskar mRNA localization and cortical anchorage of germ plasm components in the Drosophila oocyte
Source: PLoS Biol. 2021 Apr 23;19(4):e3001183. doi: 10.1371/journal.pbio.3001183 (PMC8064586; doi:10.1371/journal.pbio.3001183)
Supplement: Attachment — Submitted filename: Response to Reviewers.pdf [file pbio.3001183.s011.pdf]

We are grateful to both reviewers for their critical and constructive comments to improve our manuscript. According to reviewers' comments, we have performed additional experiments and thoroughly revised our manuscript.

We have now performed two additional experiments in response to the reviewers' comments:

1. We examined posterior localization of YFP-Vasa in *yolkless* hemizygous oocytes (*yl^16-2/Df(1)KA9* or *yl^16-6/Df(1)KA9*), and confirmed that the loss of *yolkless* is causative of the phenotype. We show that, similarly to *yl^16-2/yl^16-6* oocytes, YFP-Vasa failed to accumulate to the posterior cortex of the *yolkless* hemizygous oocyte.
2. We performed quantitative analyses of cortical localization of Staufen and Oskar at the posterior region of the oocyte. Intensity plots of Staufen and Oskar show that, in contrast to wild-type oocytes, they were spread away from the posterior cortex to more interior region of the ooplasm in mutant oocytes for *yolkless* and its ligands.

We have also substantially revised the Discussion based on the reviewers' comments. Two major revisions are as follows:

1. We have discussed the putative interaction domain of Yolkless with long Oskar.
2. We have discussed possible links between the yolk uptake process, the actin organization, the posterior microtubule organization, and the Kinesin-dependent cytoplasmic streaming.

We realized that we used transgenic flies expressing YFP-Vasa, but not GFP-Vasa, in our experiments. Thus, we have thoroughly corrected descriptions regarding YFP-Vasa in the revised manuscript (Fig 2, the Results, and the Materials and methods).

Our point-by-point responses to each comment by the reviewer follow on the next pages (the original reviewers' comments are in black and our response follows in blue text).

Reviewer 1:

The manuscript by Tanaka et al. describes parts of the mechanism that links Drosophila posterior patterning to yolk uptake into the oocyte. One of the two Oskar (Osk) isoforms, short Osk, recruits the germplasm factors to the posterior region of the oocyte, and the other one, long Osk, attaches to the surface of endosomes at the posterior cortex and is needed for the tight posterior anchoring of the germplasm factors. At the same time, localized Osk also stimulates local endocytosis. Tanaka and colleagues now convincingly show that endocytosis of yolk proteins through the Yolkless (Yl) receptor is needed for this anchoring and for establishing the proper cytoskeletal structures in the posterior region. Yl usually imports the three yolk proteins Yp1-3. Lack of yl has the strongest effect on posterior yolk import, anchoring of posterior factors, and organization of the posterior cytoskeleton. Similarly, but apparently less efficiently, disrupting in the same animal all three Yp1-3 genes also affected the posterior anchoring of long Osk and the posterior patterning. Additionally, the authors found evidence that Apolipophorin polypeptides are imported with the yolk proteins, too, and they might contribute to this process similar to the yolk proteins Yp1-3. Their functional importance is, however, not entirely clear yet.

By using ectopic (anterior) localization of tagged Osk isoforms the authors can demonstrate nicely what long Osk is capable of doing. The isolation of the complex components looks impressive and it gave them a good lead for their further, functional studies, which they performed also with mutants in the yl gene and in the Yp1-3 genes. The yl mutant generally produced a stronger phenotype than the Yp1-3 triple mutant, which is consistent with there being at least one additional protein that uses the yl receptor and contributes to the patterning function of yl. This could be Apolipophorin. However, because the gene encoding it is essential, this was not tested in the same way as for the Yp1-3 genes. Nevertheless, the model regarding Apolipophorin presented by the authors is consistent with the presented results. What was missing from the presented results with the yl and the Yp1-3 mutants are tests that show whether the mutant phenotypes are indeed caused by the known mutation in the respective genes and not due to second site hits. Admittedly, from the data, there is little doubt about this, and introducing additional genetic elements (like rescue constructs) could be a difficult task. Have other attempts been tried like using deficiencies or RNAi constructs? Even in cases where two different lines were used (e.g. yl16-2/yl16-6), the genetic background of the two alleles is probably at least very similar.

We thank for the valuable suggestion. Accordingly, we have now provided data that show the phenotype of hemizygotes for *yolkless*-null (*yl^16-2*/*Df(1)KA9* or *yl^16-6*/*Df(1)KA9*). They showed defects in the posterior localization of YFP-Vasa. This data supports our conclusion that the loss of *yolkless* is causative of the phenotype. These results are shown in new S3 Fig.

Results/Discussion:

The results might suggest that endocytosis acts by producing higher numbers of Yl receptors on endosomes (either a higher concentration of Yl on endosomes or more endosomes) and that these receptors help anchoring sufficient posterior factors. Could this be added to the discussion? It would also be good to know to which Yl domain large Osk attaches. Considering the expected topology of Yl in endosome membranes, does this interaction site support the presented model?

According to the reviewer’s suggestion, we have added a statement that "Our findings further suggest that an amount of Yl on endosomes might be critical for the proper actin remodeling to anchor germ plasm components to the oocyte cortex." to the Discussion on lines 387-389 of the revised manuscript.

Regarding the Yolkless-long Oskar interaction, we have added discussion for the putative interaction domain of Yolkless with long Oskar as follows: "Yl was associated with long Osk presumably on endocytic vesicles (Fig 1). Yl is a single-pass transmembrane protein with a large extracellular domain that binds to yolk proteins [28] (S2A Fig). The cytoplasmic tail of Yl contains two endocytic sorting signals: the noncanonical FXNPXA sorting sequence and the atypical dileucine sequence, which are recognized by Ced-6 and the AP-2 complex, respectively [27]. Ced-6 and the AP-2 complex act as clathrin adaptors and redundantly promote the endocytosis of Yl [27]. Given that long Osk is produced in the cytoplasm, it would associate with the cytoplasmic tail of Yl. It is an interesting future issue to examine whether the endocytic sorting signals and their binding adaptors in Yl are involved in the long Osk association and function." in the Discussion on lines 369-377 of the revised manuscript.

With good arguments, the authors make their case for the potential importance of the endosomes as docking sites supporting microfilament formation (although Fig 4R' seems to show as much or even more, but diffuse, F-actin than 4P'). However, could there also be a connection to kinesin-dependent cytoplasmic streaming? What are the connections between the posterior microtubule organization defect and the actin defect and how is this possible interaction affected by the mutants described here? Could this be discussed as well?

We fully agree that it is an important point to discuss possible links between the yolk uptake process, the actin organization, the posterior microtubule organization, and the kinesin-dependent cytoplasmic streaming. Accordingly, we have carefully discussed it as follows: "The posterior microtubule organization requires the plus-end-directed microtubule motor Kinesin. The Kinesin transports not only *osk* mRNA but also Dynactin to the posterior pole of the oocyte, which stabilizes the microtubule plus ends [55]. In addition, Kinesin drives bulk movement of the oocyte cytoplasm during stages 10b to 12, known as ooplasmic streaming, which is accompanied by the formation of loose and dynamic parallel microtubule arrays at the oocyte cortex [56, 57]. While the ooplasmic streaming is essential for the posterior accumulation of germ plasm components in the oocyte at the late stages of oogenesis,

its premature onset disrupts the polarized microtubule arrays along the anterior-posterior axis, resulting in defects in the oocyte axis formation and the germ plasm assembly. Previous reports indicate that the ooplasmic actin mesh assembled by Capu and Spir acts as a negative regulator of Kinesin-dependent ooplasmic streaming, probably by inhibiting the recruitment of Kinesin to the microtubule [58, 59]. Mutant oocytes for *capu* and *spir* lack the actin mesh, and show precocious onset of Kinesin-dependent ooplasmic streaming and cortical microtubule array formation at stage 8 [56-60]. Given that Capu- and Spir-mediated cortical actin remodeling was defective in $yl^{-/-}$ and Yl ligand-depleted oocytes (Fig 4), the ooplasmic actin mesh might be also disorganized by the malfunctioning of yolk uptake, resulting in microtubule disorganization at the posterior pole of the oocyte." in the Discussion on lines 414-432 of the revised manuscript.

A possibly interesting aspect of this work that might deserve some additional consideration and discussion is that this mechanism couples nutrient uptake with evolutionary fitness. Insufficient yolk uptake not only slows down development and reduces the hatching rate, but it also reduces the number of germline progenitors that are being formed at the posterior end (this is all shown in this manuscript). This mechanism might thus help to adjust fecundity to food availability.
According to the reviewer's suggestion, we have added a statement that "In addition, we found that the yolk depletion caused reduction in the number of pole cells in stage-5 embryos (Fig 2G and S1 Data), suggesting that there is a mechanism that might adjust fecundity to food availability." to the Discussion on lines 365-367 of the revised manuscript.

Minor issues and typos:
In Figure 3A-F, it is not clear what the white posterior crescent signal reflects (probably Kin-B-Gal). Could a different LUT be used? With the exception of E, these panels are difficult to judge.
According to the reviewer's suggestion, images for Staufen and Kin-βGal have been presented separately in addition to merged images in new Fig 3A-3F.

line 295: Fig 4A-B(-C): only the lateral and posterior regions display a reduction of FM4-64 signal. Not the anterior.
We agree that a reduction of FM4-64 signal at the oocyte anterior was unclear in mutants. Accordingly, we have changed the statement as follows: "We found that the loss of Yl or the depletion of Yl-ligands led to reduction of FM4-64 signals <u>at the lateral and posterior regions in the oocyte</u> (Fig 4B and 4C)," in the Results on lines 277-279 of the revised manuscript.

line 134: "This result is consistent…" is an isolated statement, and in the context of the RNA binding proteins just described to bind to Osk, it does not support a direct binding of Oskar to RNA.

According to the reviewer’s suggestion, we have rewritten the sentence as "Given that Osk interacts directly with RNAs [20, 21], it might be associated to these proteins via RNAs." on lines 122-123.

Fig S2B: state what blue sequence means (see D, E)

The information has provided on lines 746-747.

line 238: "is probably caused" sounds too speculative and could be replaced by "might be caused"

As suggested, we have replaced "is probably caused" with "might be caused" on line 222.

line 381: completed (since "developed" on line 382)

As suggested, we have corrected the tense as "completed" on line 359.

line 411: rather than by/through a general decrease in (missing word)

As suggested, we have replaced "rather than" with "rather than by" on line 386.

Reviewer 2:

In this manuscript Tanaka et al. identify the yolk receptor, Yolkless, as protein that is pulled-down with the long isoform of Oskar. They go onto show that yolkless mutants cause similar defects in the anchoring of the pole plasm and the organisation F-actin and microtubule cytoskeletons to the loss of this long Oskar isoform. They also identify apolipoprotein 1 as a novel yolk protein in Drosophila and show that removal of all yolk proteins and 50% of apolipoprotein 1 gives a similar phenotype to loss of yolkless. Thus, these results reveal an unexpected link between vitellogenesis and the formation of the pole plasm.

The data are well-presented and generally support the main conclusions, but the phenotype is rather subtle. Staufen and Oskar are less tightly localised to the posterior cortex and the yolk protein null; apol1 heterozygotes show a 30% reduction in the number of pole cells. Although the involvement of yolk endocytosis in polar plasm anchoring is an interesting twist, there are a number of issues that will need to be resolved before publication and I suspect that this work is more appropriate for PLoS Genetics than PLoS Biology.

Main points:

1) The localisation phenotypes need to be properly quantified. For example, the main text states that "We found that, in yl-/- oocytes, Stau did not thoroughly target to the posterior pole and was observed distant from the posterior cortex, resulting in diffuse localization of Osk", but Figures 2 and 3 do not show this. In Fig 3H, almost all Staufen forms a clear posterior crescent, with a slight haze in the cytoplasm. Although Oskar protein is slightly more diffuse in Figure 2I-M, it is still all very close to the posterior cortex and cannot accurately be described as "diffuse". Similarly "Stau also failed to target to the posterior pole of these oocytes" is a gross overstatement of the data shown in Figure 3 and H. What is needed is a plot of the distributions of Staufen and Oskar across the anterior-posterior axes of multiple oocytes.

We thank for the valuable suggestion and agree that it is important to assess the cortical localization of Staufen and Oskar at the posterior region of the oocyte. Accordingly, we performed quantitative analyses of cortical localization of immunostained Staufen and Oskar at the oocyte posterior in wild type, *yl*$^{-/-}$, and yolk protein-depleted mutants. The data show that both Staufen and Oskar were significantly diffused from the posterior cortex in mutant oocytes for *yolkless* and its ligands. These results are now shown in new Fig 2N, 2U, and S1 Data. In addition, new images for Staufen and Oskar at stage 9 and 10b have shown in new Fig 2H-2M and Fig 2O-2T. Concomitantly, we have omitted images for Staufen from new Fig 3G-3I because diffused localization of Staufen was obscure.

2) Figure S5 is misleading as it implies that Yolk endocytosis is upstream of microtubule alignment

and oskar mRNA localisation. The microtubules are normal initially and yl mutants only disrupt the maintenance of this organisation.

We thank for pointing it out. Accordingly, we have rewritten the sentence as "Maintenance of microtubule alignment" in a schematic model of new S6 Fig.

3) The localisation of kinesin-betaGal to the centre of the oocyte in Fig3E seems anomalous given that Staufen is pretty well localised, but it is hard to see kinesin-betaGal is also enriched at the posterior. This is another phenotype that would benefit from proper quantification.

As mentioned above, we performed quantitative analyses of cortical localization of Staufen and Oskar at the posterior region of the oocyte and show that mutant oocytes for *yolkless* and its ligands were defective in tight cortical localization of Staufen and Oskar. We have also modified Fig 3A-3F in which images for Staufen and Kin-βGal are presented separately to clearly display the diffused localization of Staufen. We believe that these data have strengthened the manuscript.
